# Atomically synergistic Zn-Cr catalyst for iso-stoichiometric co-conversion of ethane and CO_2_ to ethylene and CO

**DOI:** 10.1038/s41467-024-44918-8

**Published:** 2024-01-30

**Authors:** Ji Yang, Lu Wang, Jiawei Wan, Farid El Gabaly, Andre L. Fernandes Cauduro, Bernice E. Mills, Jeng-Lung Chen, Liang-Ching Hsu, Daewon Lee, Xiao Zhao, Haimei Zheng, Miquel Salmeron, Caiqi Wang, Zhun Dong, Hongfei Lin, Gabor A. Somorjai, Fabian Rosner, Hanna Breunig, David Prendergast, De-en Jiang, Seema Singh, Ji Su

**Affiliations:** 1 Energy Storage and Distributed Resources Division, Lawrence Berkeley National Laboratory, Berkeley, CA USA; 2 The Molecular Foundry, Lawrence Berkeley National Laboratory, Berkeley, CA USA; 3 Department of Chemistry, University of California, Riverside, CA USA; 4 Materials Sciences Division, Lawrence Berkeley National Laboratory, Berkeley, CA USA; 5 Sandia National Laboratories, Livermore, CA US; 6 National Synchrotron Radiation Research Center, Science-Based Industrial Park, Hsinchu, Taiwan; 7 Department of Soil and Environmental Sciences, National Chung Hsing University, Taichung, Taiwan; 8 Gene and Linda Voiland School of Chemical Engineering and Bioengineering, Washington State University, Pullman, WA USA; 9 Department of Chemistry, University of California, Berkeley, CA USA; 10 Energy Analysis and Environmental Impacts Division, Lawrence Berkeley National Laboratory, Berkeley, CA USA; 11 Department of Chemical and Biomolecular Engineering, Vanderbilt University, Nashville, TN USA

**Keywords:** Heterogeneous catalysis, Materials for energy and catalysis

## Abstract

Developing atomically synergistic bifunctional catalysts relies on the creation of colocalized active atoms to facilitate distinct elementary steps in catalytic cycles. Herein, we show that the atomically-synergistic binuclear-site catalyst (ABC) consisting of ${{{{{\rm{Zn}}}}}}^{\delta+}$-O-Cr^6+^ on zeolite SSZ-13 displays unique catalytic properties for iso-stoichiometric co-conversion of ethane and CO_2_. Ethylene selectivity and utilization of converted CO_2_ can reach 100 % and 99.0% under 500  °C at ethane conversion of 9.6%, respectively. In-situ/ex-situ spectroscopic studies and DFT calculations reveal atomic synergies between acidic Zn and redox Cr sites. ${{{{{\rm{Zn}}}}}}^{\delta+}$ ($0 \, < \, \delta \, < \, 2$) sites facilitate β-C-H bond cleavage in ethane and the formation of Zn-H^*δ*-^ hydride, thereby the enhanced basicity promotes CO_2_ adsorption/activation and prevents ethane C-C bond scission. The redox Cr site accelerates CO_2_ dissociation by replenishing lattice oxygen and facilitates H_2_O formation/desorption. This study presents the advantages of the ABC concept, paving the way for the rational design of novel advanced catalysts.

## Abstract (web summary)

Designing catalysts with atomically synergistic sites shows great promise as a pathway for advanced catalyst development. Here the authors report Zn-Cr catalyst with atomically binuclear active sites for iso-stoichiometric co-conversion of ethane and CO_2_ through proximal atomic synergy.

## Introduction

The synergistic effects generated by mixed or supported bifunctional catalysts are often claimed to promote the catalytic performance of traditional heterogeneous catalysts^1–9^. Recently, the construction of synergistic pair-sites with colocalized metal atoms to facilitate distinct elementary steps in the catalytic reaction has been established as a crucial step toward atomically synergistic bifunctional catalyst development^10–12^. The interaction between these adjacent metal atoms, similar to the metal-support interaction of traditional heterogeneous catalysts^3,13^, offers the possibility to modulate their respective electronic structure to further enhance their catalytic activity^11,14–17^. However, precisely controlling their colocalization requires a complicated synthesis process with multiple synthesis steps to load single atoms^10,18^. Moreover, the stability of the well-defined synergistic sites is debatable, especially under harsh reaction conditions such as high reaction temperatures^18,19^. The benefits and remaining challenges of atomically synergistic bifunctional catalysts motivate us to further explore new synthesis routes, new reaction applications, and their synergistic effects with the goals to accelerate the development of next-generation atomically synergistic catalysts.

Growing CO_2_ emissions and abundant shale gas reserves have prompted a significant amount of research to explore efficient approaches for co-utilizing the products to produce value-added chemicals^20–22^. Ethane, the second-largest component of shale gas, is an ideal alternative hydrogen source for CO_2_ conversion. The co-conversion of ethane and CO_2_ (C_2_H_6_ + CO_2_ → C_2_H_4_ + CO + H_2_O) is a viable alternative to the ethane steam cracking for ethylene production under the goal of net negative CO_2_ emissions^21^. Furthermore, iso-stoichiometric co-conversion of ethane and CO_2_ (ICEC) to ethylene and CO is crucial for direct downstream processes such as the hydroformylation reaction to produce aldehyde^23^ and polymerization process to produce polyketones^24–27^. However, the ICEC process lacks a viable catalyst for achieving high ethylene selectivity and CO_2_ utilization simultaneously. Metal-based catalysts suffer from the inevitable cleavage of C-C bonds through dry reforming pathways and lower the ethylene selectivity^22,28–30^. Oxide-based catalysts exhibit the great merit of preferential C–H bond scission over C–C bond scission pathways but require excessive CO_2_ cofeeding to reduce ethane adsorption and scavenge the surface H species^31,32^. Specifically, Zn and Cr oxide-based catalysts were widely studied for co-conversion of ethane and CO_2_. The acidic Zn^2+^ site displayed high activity for C-H bond cleavage in ethane and CO_2_ activation, requiring the participation of adjacent active sites to form binuclear sites^33–35^. The challenge is that acidified Zn^2+^-H hydride displays a capacity for C-C bond scission of ethane, leading to undesired production of methane. Redox Cr^6+^ sites require lattice oxygen as a H acceptor to dissociate C-H bonds but trigger the formation of less active Cr^3+^ species. The reoxidation of Cr^3+^ to Cr^6+^ is limited by slow O abstraction from CO_2_^20^. The above analyses thus indicate the need for the development of an atomically-synergistic binuclear Zn–O–Cr site catalyst for ICEC, with Zn facilitating CO_2_ adsorption and activation to provide O species for Cr^6+^ regeneration and adjacent Cr as an electron donor reducing the acidity of Zn^2+^ to facilitate its activity and selectivity for C-H bond scission^6,36–39^. More generally, exploring a cooperative redox and acid-base catalytic mechanism for ICEC is highly desirable.

In this work, we show the successful fabrication and demonstration of a Zn-O-Cr atomically-synergistic binuclear-site catalyst (ABC) that is highly efficient for ICEC with high ethylene selectivity and utilization of converted CO_2_ (${{{{{\rm{U}}}}}}_{{{{{{\rm{CO}}}}}}_{2}}$). As we will show, compared with pure Zn and Cr catalysts, Zn-O-Cr ABC displays ~1.5 and ~4-fold higher catalytic activity with 100% ethylene selectivity and 99.0% ${{{{{\rm{U}}}}}}_{{{{{{\rm{CO}}}}}}_{2}}$ under optimized reaction conditions. Key to this high performance is the discovery that Cr facilitates the formation of a ${{{{{\rm{Zn}}}}}}^{\delta+}$ ($0 \, < \, \delta \, < \, 2$) site to enhance the β-C-H bond cleavage of ethane, while the resulting Lewis base Zn-H^*δ*-^ hydride favors CO_2_ adsorption and activation, and prevents C-C bond scission of ethane. The redox Cr site accelerates CO_2_ dissociation and facilitates H_2_O formation/desorption. The apparent activation energies of ethane conversion and CO_2_ conversion are ~70.9 and ~74.0 kJ/mol, which demonstrates the rate matching achieved in ICEC.

## Results

### Controlling Zn and Cr coordination structure

**Fig. 1:**
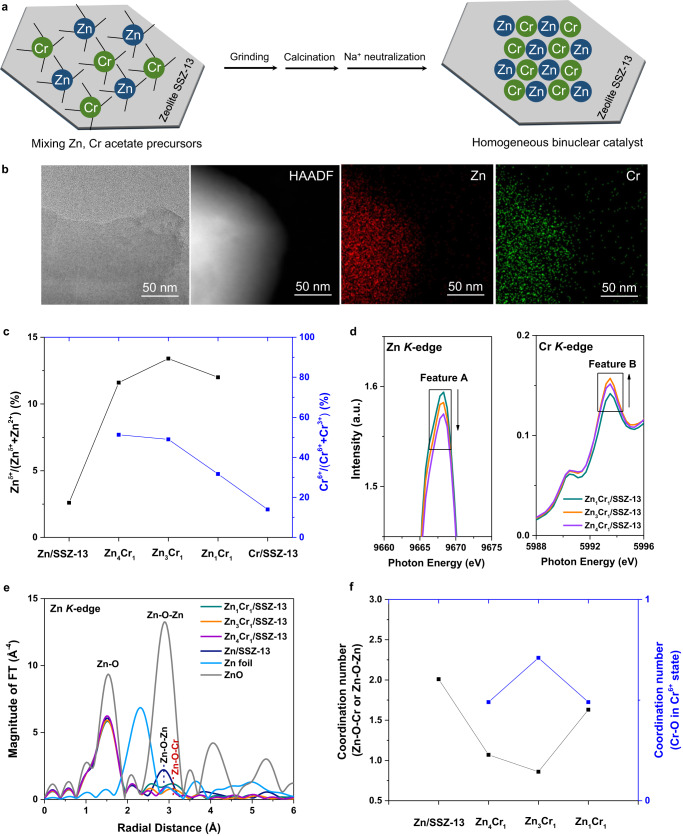
Coordination of Zn-O-Cr sites in atomically-synergistic binuclear-site catalyst (ABC). **a** Schematic illustration of the fabrication process of Zn-O-Cr ABC. **b** Representative TEM and HAADF-STEM images and EDS mapping of Zn-O-Cr ABC with Zn/Cr ration of 3/1; **c** The proportion of Zn^*δ*+^ $(0 \, < \, \delta \, < \, 2)$ (left) and Cr^6+^ (right) with varying Zn/Cr ratios (Results derived from Auger spectra of Zn *LMM* and Cr 2*p*_3/2_ XPS spectra); **d** Enlarged electron transition features in Zn and Cr *K-edge* XANES spectra; **e** *k*^3^-weighted Fourier-transformed extended X-ray absorption fine structure (FT-EXAFS) spectra (Zn *K-edge*) of Zn-O-Cr ABCs with varying Zn/Cr ratios, with Zn foil and ZnO as references; **f** Coordination number (CN) of Zn-O-Cr(Zn) and Cr-O in Cr^6+^ state in Zn-O-Cr ABCs with varying Zn/Cr ratios.

Catalysts were synthesized by mixing a constant total amount of zinc (II) acetate and chromium (III) acetate hydroxide on a SSZ-13 zeolite support with varying Zn/Cr molar ratios, followed by direct decomposition at 550°C and then Na^+^ neutralization of acidic site on supports (Fig. 1a). The catalyst synthesis method developed here will be referred to as the dry-deposition method (The experimental details are summarized in Methods). The catalysts are denoted as Zn_*x*_Cr_*y*_/SSZ-13, where *x/y* refers to the ratio of Zn/Cr (*x* = 1, *y* = 0; *x/y* = 1/2, 1, 2, 3, 4; *x* = 0, *y* = 1). The textural properties, composition analysis, and surface acidity of SSZ-13 and synthesized catalysts are characterized by N_2_ physisorption, X-ray fluorescence (XRF), and attenuated total reflection Fourier transform infrared (ATR-FTIR) measurements (Supplementary Figs. 1-2 and Tables 1-2). Transmission electron microscopy (TEM), scanning TEM (STEM) images, and energy dispersive spectroscopy (EDS) elemental mappings (Fig. 1b and Supplementary Figs. 3-5) demonstrate high dispersion of Zn and/or Cr oxide phases, with no observable sintering of oxide nanoparticles. The X-ray diffraction (XRD) results reveal the absence of spinel ZnCr_2_O_4_ and zincite ZnO for all dry-deposition synthesized catalysts. Cr_2_O_3_ phases with R3c space group were only observed in Cr/SSZ-13 and Zn_1_Cr_2_/SSZ-13 (Supplementary Fig. 6). The control samples with same amount Zn and Cr precursors were prepared via a co-precipitation method (CP), followed by Na^+^ neutralization. ZnCrO_x_ nanoparticles are observed in CP-synthesized samples (Supplementary Fig. 7). And a phase transition from spinel ZnCr_2_O_4_ to ZnO (Supplementary Fig. 8) was detected on CP-synthesized catalysts with Zn/Cr ratios varying from 1/2 to 3/1^40–42^.

Figure 1c, Supplementary Figs. 9-11 and Supplementary Table 3 show X-ray photoelectron spectroscopy (XPS) results for Zn 2*p*, Zn *LMM* Auger and Cr 2*p*_3/2_ spectra. The formation of Zn^*δ*+^ (0 < *δ* < 2) was confirmed through Auger spectra of Zn *LMM*. The subpeaks at 987.5 and 990.0 eV in Auger spectra of Zn *LMM* (Supplementary Fig. 10) are assigned to the Zn^2+^ and ${{{{{\rm{Zn}}}}}}^{\delta+}$ ($0 \, < \, \delta \, < \, 2$), respectively;^43^ And the subpeaks at ~576 and ~580 eV in Cr 2*p*_3/2_ XPS (Supplementary Fig. 11) were assigned to the Cr^3+^ and Cr^6+^, correspondingly^44^. Figure 1c shows the proportion of ${{{{{\rm{Zn}}}}}}^{\delta+}$ 0 < *δ* < 2 and Cr^6+^ relative to the total amount of $({{{{{\rm{Zn}}}}}}^{\delta+}+{{{{{\rm{Zn}}}}}}^{2+})$ and (Cr^6+^+Cr^3+^) in Zn_*x*_Cr_*y*_/SSZ-13 catalysts, respectively. These proportions vary with Zn/Cr ratios, with the highest Zn^*δ*+^(0 < δ < 2) proportion generated at Zn/Cr ratio of 3/1 and the highest Cr^6+^ proportions produced at Zn/Cr ratios of 3/1 and 4/1. We hypothesize that the proximal electronic interactions between Zn and Cr could modify their respective oxidation states. This is consistent with the Zn *L*_3_-edge X-ray absorption near-edge structure (XANES) spectra analyses (Supplementary Fig. 12), which suggests a decrease in the oxidation state of Zn in Zn_*x*_Cr_*y*_/SSZ-13 samples compared to pure Zn/SSZ-13 sample. The ${{{{{\mathrm{Zn}}}}}}^{\delta+}$ and Cr^6+^ content in the CP-synthesized Zn_3_Cr_1_/SSZ-13 sample is similar to those of pure Zn/SSZ-13 and Cr-SSZ-13 samples, respectively (Supplementary Fig. 13).

XANES spectra (Fig. 1d and Supplementary Fig. 14) and extended X-ray absorption fine structure (EXAFS) spectra (Fig. 1e, f, Supplementary Figs. 15–18, Supplementary Tables 4-5) were used to study coordination structures of Zn and Cr sites. As shown in Fig. 1d and Supplementary Fig. 14, the electron transition from Zn 1*s* to Zn 4*p* unoccupied orbitals (Feature A) is revealed in Zn *K*-edge XANES spectra; and Cr *K*-edge XANES spectra exhibits the peak of electron transition from Cr 1*s* to Cr 3*d*-O 2*p* unoccupied orbitals (Feature B). As the Zn/Cr ratio increases from 1/1 to 4/1 in Zn_x_Cr_y_/SSZ-13 catalysts, the intensity of feature A decreases, suggesting electron occupation in Zn 4*p* unoccupied orbitals, while the intensity of feature B increases, which suggests electrons transfer out of Cr 3*d*-O 2*p* orbitals near the conduction band minimum. We hypothesize a link between these observations, that electronic charge transfers from Cr 3*d*-O 2*p* character in the conduction band to Zn 4*p*^45,46^, due to a strong Zn-Cr interaction. Thus, the charge transfer decreases the oxidative state of Zn^2+^ to ${{{{{\rm{Zn}}}}}}^{\delta+}$ ($0 \, < \, \delta \, < \, 2$), which is consistent with XPS and Auger results (Fig. 1c). In Fig. 1e, the scattering peaks at 1.50 Å are assigned to Zn-O coordination in the first shell. The pure Zn/SSZ-13 and Zn_*x*_Cr_*y*_/SSZ-13 samples exhibited a significantly lower intensity for this Zn-O scattering peak than the ZnO reference, suggesting a higher degree of crystal disorder^47,48^. The scattering peaks at ~2.85 Å are assigned to Zn-O-Zn coordination in the second shell. Compared to Zn/SSZ-13 and the ZnO reference, Zn_1_Cr_1_/SSZ-13 and Zn_3_Cr_1_/SSZ-13 exhibited no peak for Zn-O-Zn coordination. Instead, a new peak at ~3.08 Å should be attributed to the Zn-O-Cr bond^41^. Differently, Zn_4_Cr_1_/SSZ-13 sample has both Zn-O-Cr and Zn-O-Zn bonds. Cr *K*-edge Fourier transformed EXAFS (FT-EXAFS) spectra of Cr-containing samples (Supplementary Fig. 15) displayed Cr-O scattering peaks located at 1.51 Å, which is similar to standard peak of Cr-O scattering 1.50 Å in Cr^III^_2_O_3_; but as Zn/Cr ratio increase to 3/1 and 4/1, a visible shoulder peak corresponding to standard peak of Cr-O scattering at 1.20 Å in K_2_Cr^VI^_2_O_7_ appeared, indicating that Zn_3_Cr_1_/SSZ-13 and Zn_4_Cr_1_/SSZ-13 samples have two types of Cr-O coordination. Supplementary Tables 4-5 show the EXAFS-derived fitting parameters. Zn_*x*_Cr_*y*_/SSZ-13 catalyst (*x/y* = 1, 3, 4) samples show Zn-O-Cr coordination with a bond distance ~3.4 Å in phase-corrected space, which corresponds to the emergent peak at ~3.08 Å in Fig. 1e^41^. These results provide solid evidence for the formation of a Zn^*δ*+^-O-Cr^6+^ structure in Zn_*x*_Cr_*y*_/SSZ-13. Notice that Zn_*x*_Cr_*y*_/SSZ-13 samples also exhibited lower coordination numbers (CNs) for Zn-O and Zn-O-Cr compared to the pure Zn/SSZ-13 sample (Fig. 1f and Supplementary Table 4), which results from Cr colocalization. Especially, for the Zn_3_Cr_1_/SSZ-13 and Zn_4_Cr_1_/SSZ-13 samples, the CNs of Zn-O and Zn-O-Cr are 3.94 and 0.86, and 3.77 and 1.07, respectively. And Zn_3_Cr_1_/SSZ-13 has the highest CN for the Cr-O bond in hexavalent (Cr^6+^) states. Therefore, these results confirm the formation of hetero binuclear Zn^*δ*+^-O-Cr^6+^ sites, and Zn_3_Cr_1_/SSZ-13 exhibits the highest population of the Zn^*δ*+^-O-Cr^6+^ site.

### Catalytic performance of Zn-O-Cr ABC for ICEC

**Fig. 2:**
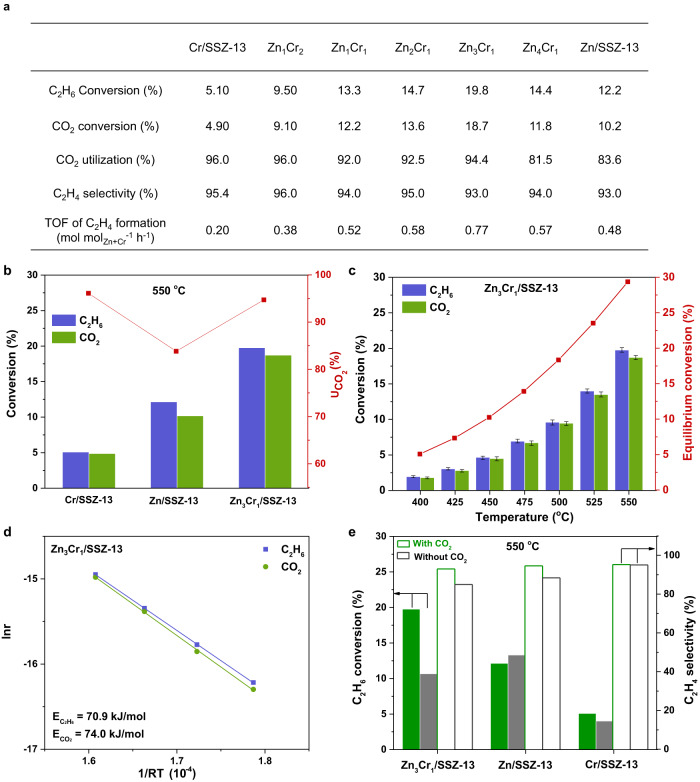
Iso-stoichiometric co-conversion of ethane and CO_2_ (ICEC). **a** Catalytic performance of Zn-O-Cr ABCs (Reaction condition: Temperature = 550  °C; Reactant composition = 5% C_2_H_6_ + 5% CO_2_ + 90% Ar; WHSV = 7500 mL g^−1^_cat_ h^−1^); **b** C_2_H_6_ and CO_2_ conversion, utilization of converted CO_2_ (${{{\mbox{U}}}}_{{{{{{\rm{CO}}}}}}_{2}}$) of Cr/SSZ-13, Zn/SSZ-1_3_, and Zn_3_Cr_1_/SSZ-13 catalysts in ICEC at 550  °C; **c** Reaction temperature dependent performance of Zn_3_Cr_1_/SSZ-13 catalyst. **d** Arrhenius plots of Zn_3_Cr_1_/SSZ-13 catalyst (obtained when both C_2_H_6_ and CO_2_ conversions are <10 %). **e** C_2_H_6_ conversion and C_2_H_4_ selectivity over Zn_3_Cr_1_/SSZ-13, Zn/SSZ-13, and Cr/SSZ-13 with (green columns) /without (gray columns) CO_2_ at 550  °C.

Figure 2a, b and Supplementary Fig. 19 and Table 6 show the ICEC catalytic performance of Zn-O-Cr ABCs under the reaction conditions of weight hourly space velocity (WHSV) of 7500 mL g^−1^_cat_ h^−1^ and a CO_2_/ethane ratio of 1 at 550  °C. The Zn_3_Cr_1_/SSZ-13 ABC catalyst displayed the best performance among all tested catalysts, with the highest C_2_H_6_ conversion (19.8%), CO_2_ conversion (18.7%), turnover frequency (TOF) of C_2_H_4_ formation (0.77 mol mol_Zn+Cr_^−1^ h^−1^), as well as excellent C_2_H_4_ selectivity (93.0%) and ${{{{{\rm{U}}}}}}_{{{{{{\rm{CO}}}}}}_{2}}$ (94.4%). Previous studies have mainly focused on achieving high ethane conversion and ethylene selectivity in co-conversion of ethane and CO_2_ by co-feeding excess CO_2_ at CO_2_/ethane ratios of 2–6^49–53^. In the ICEC process the C_2_H_4_ selectivity and ${{{{{\rm{U}}}}}}_{{{{{{\rm{CO}}}}}}_{2}}$ are two important factors for evaluating the success of catalyst development. Utilization of converted CO_2_ (${{{{{\rm{U}}}}}}_{{{{{{\rm{CO}}}}}}_{2}}$) is defined as the ratio of CO_2_ conversion to ethane conversion on an iso-stoichiometric basis. Compared with previous studies in Supplementary Table 7 and Fig. 21, the Zn_3_Cr_1_/SSZ-13 catalyst displays the highest space-time yield (STY) of C_2_H_4_ formation (0.086 kg h^−1^ kg_cat_^−1^) and the highest ${{{{{\rm{U}}}}}}_{{{{{{\rm{CO}}}}}}_{2}}$ at 550 °C, which clearly shows the superiority of Zn–O–Cr ABCs. Furthermore, Fig. 2c and Supplementary Table 7 show C_2_H_4_ selectivity can achieve 100% and ${{{{{\rm{U}}}}}}_{{{{{{\rm{CO}}}}}}_{2}}$ is 99.0% under the reaction temperature of 500  °C, with ethane conversion of 9.6% and CO_2_ conversion of 9.5%. These results further demonstrate the advantage of Zn-O-Cr ABCs suitable for ICEC. In ICEC, the reaction system includes the reactants’ (ethane and CO_2_) adsorption, activation, reaction, and products’ (ethylene, CO, and H_2_O) formation, and desorption. The apparent activation energies (E_a_) for ethane conversion and CO_2_ conversion are key to evaluating the rate matching of both reactions. Figure 2d shows similar E_a_ (results were calculated based on Fig. 2c and Supplementary Fig. 22) for ethane dehydrogenation (70.9 kJ/mol) and CO_2_ hydrogenation (74.0 kJ/mol). This further demonstrates the feasibility and success of the Zn-O-Cr ABCs concept for desired ICEC catalyst development.

Figure 2a and Supplementary Fig. 19 reveal that there is a clear correlation between the proportion of Zn^*δ*+^-O-Cr^6+^ sites and ICEC performance. Pure Cr/SSZ-13 catalyst displayed slightly higher ethylene selectivity (95.4%) and higher ${{{{{\rm{U}}}}}}_{{{{{{\rm{CO}}}}}}_{2}}$ (96.0%) than Zn_3_Cr_1_/SSZ-13 sample, but has the lowest C_2_H_6_ conversion (5.1 %), CO_2_ conversion (4.9%), TOF of C_2_H_4_ formation (0.20 mol mol_Zn+Cr_^−1^ h^−1^). Increasing Zn contents in Zn-O-Cr ABCs, with Zn/Cr ratios from 1/2 to 3/1, has little effect on ethylene selectivity and ${{{{{\rm{U}}}}}}_{{{{{{\rm{CO}}}}}}_{2}}$. Instead, increased Zn contents promote higher conversion of C_2_H_6_ and CO_2_, and leads to increased TOF of ethylene formation. Further increasing the Zn content to Zn/Cr ratio of 4/1, triggers lower ethane (14.4%) and CO_2_ (11.7%) conversions, resulting in a much lower ${{{{{\rm{U}}}}}}_{{{{{{\rm{CO}}}}}}_{2}}$ (81.5 %). Pure Zn/SSZ-13 exhibited further decreased C_2_H_6_ (12.1%) and CO_2_ (10.2%) conversions, compared with Zn_4_Cr_1_/SSZ-13. Therefore, we hypothesize that the Zn^*δ*+^ in the binuclear site of Zn^*δ*+^-O-Cr^6+^ is the primary active site for C_2_H_6_ dehydrogenation. Interestingly, the Zn^*δ*+^ proportion also displays a correlation with CO_2_ conversion, which indicates that the Zn^*δ*+^ sites are also involved in CO_2_ adsorption, activation, or reaction. In previous studies, binuclear Zn-O-Zn catalysts have been reported to have a high activity in ethane or propane dehydrogenation, but lack the capacity of efficient CO_2_ activation, leading to insufficient CO_2_ utilization^35,54,55^. In our study, the Zn_3_Cr_1_/SSZ-13 ABC with the highest amount of Zn^*δ*+^-O-Cr^6+^ sites displayed ~1.5 and ~4-fold higher ethane dehydrogenation and CO_2_ conversion performance than pure Zn and Cr catalysts, respectively (Fig. 2b), which indicates the Cr^6+^ site of Zn^*δ*+^-O-Cr^6+^ is also involved in the activation and reaction of C_2_H_6_ and CO_2_. From the above analyses, we conclude that the unique performance of Zn-O-Cr ABCs for ICEC relies on the atomic synergies within the Zn^*δ*+^-O-Cr^6+^ site.

To study the atomic synergies between Zn^*δ*+^-O-Cr^6+^ site in ICEC, we compared the C_2_H_6_ dehydrogenation performance of Zn_3_Cr_1_/SSZ-13 with pure Zn/SSZ-13 and Cr/SSZ−13 samples in the presence and absence of CO_2_. In Fig. 2e, we found the CO_2_ co-feeding significantly improved C_2_H_6_ conversion (19.8% vs. 10.7%) and C_2_H_4_ selectivity (93.0% vs. 85.0%) for Zn_3_Cr_1_/SSZ-13, with a high ${{{{{\rm{U}}}}}}_{{{{{{\rm{CO}}}}}}_{2}}$ up to 94.4%. By contrast, for Zn/SSZ−13, CO_2_ addition fails to enhance its C_2_H_6_ conversion (12.1% vs. 13.3%) but results in an increase in C_2_H_4_ selectivity (94.6% vs. 86.0%). The higher C_2_H_4_ selectivity is due to competitive adsorption of CO_2_ over C_2_H_6_, preventing the ethane cracking reaction, which has been reported previously^56^. Compared with Zn/SSZ−13, the improved C_2_H_6_ conversion of the Zn_3_Cr_1_/SSZ-13 catalyst is due to the generation of Zn^*δ*+^ in Zn^*δ*+^-O-Cr^6+^. For the Cr/SSZ−13 sample, CO_2_ introduction results in a higher C_2_H_6_ conversion (5.1% vs 4.0%) with a high ${{{{{\rm{U}}}}}}_{{{{{{\rm{CO}}}}}}_{2}}$ of 96%, but it did not change the C_2_H_4_ selectivity (95.4% vs 95.1%). Cr-based catalysts are reported to catalyze CO_2_ and ethane conversion through the redox (or MvK) mechanism^20^. CO_2_ introduction could favor lattice oxygen replenishment to regenerate highly reactive Cr^6+^ species and shift the reaction equilibrium of C_2_H_6_ dehydrogenation, thus leading to a higher activity. These results indicate that a reaction synergy between ethane and CO_2_ conversions could only occur at the atomically synergistic Zn^*δ*+^-O-Cr^6+^ site.

To further validate the superiority of Zn-O-Cr ABCs, we compared the performance of Zn_3_Cr_1_/SSZ−13 prepared by the dry-deposition method with the sample synthesized by the traditional co-precipitation (CP) method. Supplementary Fig. 23 shows that the dry-deposition synthesized Zn_3_Cr_1_/SSZ-13 catalyst exhibited >4-fold higher ethane conversion (19.8% vs 4.5%) and higher ${{{{{\rm{U}}}}}}_{{{{{{\rm{CO}}}}}}_{2}}$ (94.4% vs 83.2%). The CP-synthesized Zn_3_Cr_1_/SSZ-13 sample was found to contain large Zn/Cr oxide particles and separate ZnCr_2_O_4_ and ZnO phases, resulting in loss of the atomic synergies of the Zn^*δ*+^-O-Cr^6+^ site, which we correlate with its poor performance. We also studied the stability and regeneration ability of the Zn_3_Cr_1_/SSZ-13 ABC catalyst. Supplementary Fig. 24 shows that the ethylene selectivity and ${{{{{\rm{U}}}}}}_{{{{{{\rm{CO}}}}}}_{2}}$ remained nearly 100% during a total of 150 h in 3 cycles. And the decayed conversion of ethane and CO_2_ can be totally regenerated by oxidative treatment in air at 500 °C. This excellent durability and regeneration ability demonstrates the structural stability of Zn^*δ*+^-O-Cr^6+^ site.

### Electronic structure of binuclear Zn^*δ*+^-O-Cr^6+^ sites during the reaction

**Fig. 3:**
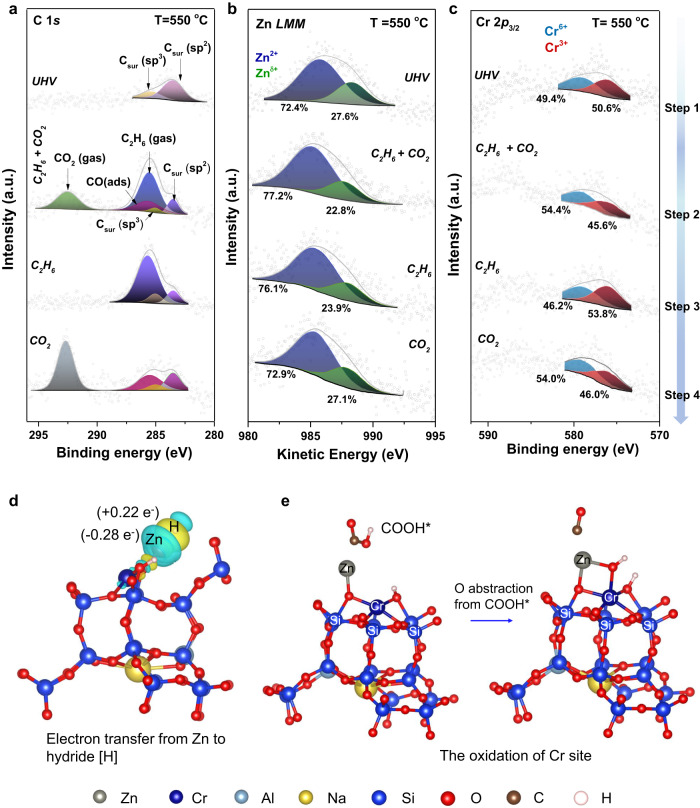
Electronic structure changes of binuclear Zn^*δ*+^-O-Cr^6+^sites. In situ ambient pressure X-ray photoelectron spectroscopy (APXPS): **a** C 1*s* spectra, **b** Auger spectra of Zn *LMM*, and **c** Cr 2*p*_3/2_ spectra as a function of reaction conditions for Zn_3_Cr_1_/SSZ-13. T = 550  °C: ultra-high vacuum (UHV), C_2_H_6_ (50 mTorr) + CO_2_ (50 mTorr), C_2_H_6_ (100 mTorr), and CO_2_ (100 mTorr) in sequence. **d** Electron transfer from Zn to H after the 2^nd^ C-H bond scission in ethane (H: white; Zn: gray), where electron accumulation and depletion are represented by yellow (Δρ = +1 × 10^−3^ e bohr^−3^) and cyan (Δρ = −1 × 10^−3^ e bohr^−3^) respectively. **e** The oxidation of Cr in Zn-O-Cr when decomposing COOH* intermediate.

In situ ambient pressure X-ray photoelectron spectroscopy (APXPS) was employed to examine the electronic structure of binuclear Zn^*δ*+^-O-Cr^6+^ sites and study the atomic synergies between Zn^*δ*+^ and Cr^6+^ sites in ICEC. Figure 3a–c shows ambient pressure spectra indicating C 1*s* binding energies, Zn *LMM* Auger kinetic energies, and Cr 2*p*_3/2_ binding energies.

Step 1: Zn_3_Cr_1_/SSZ-13 was first tested under 550 °C in an ultra-high vacuum (UHV). The temperature of 550 °C was used to match the reaction temperature in Fig. 2. In Auger spectra of Zn *LMM* (Fig. 3b), both Zn^2+^ (dark blue) and Zn^*δ*+^ (green) species were detected with a percentage distribution of 72.4% and 27.6%, respectively. In Fig. 3c, Cr^3+^ (dark red) and Cr^6+^ (light blue) were detected with a proportion of 50.6% and 49.4%, respectively.

Step 2: Then the Zn_3_Cr_1_/SSZ-13 was subjected to simulated reaction conditions of ICEC by co-feeding 50 mTorr C_2_H_6_ and 50 mTorr CO_2_ under 550 °C. The signals of gaseous C_2_H_6_ and CO_2_ were detected in Fig. 3a. Notably, adsorbed CO species (CO_ads_) at 285.5 eV were observed^57,58^, which indicates the ICEC reaction occurred. Under the reaction conditions, the proportion of Zn^*δ*+^ decreased (from 27.6% to 22.8%) and Cr^6+^ ratio increased (from 49.4% to 54.4%). DFT calculations (Fig. 3d) indicate that the oxidation from Zn^*δ*+^ to Zn^2+^ is due to the formation of Zn-H^*δ*-^ hydride during the ethane dehydrogenation, with more negative charge accumulated on H^*δ*-^. The oxidation of Cr^3+^ to Cr^6+^ may result from lattice oxygen replenishment via CO_2_ dissociation^20^. These results indicate both Zn^*δ*+^ and Cr^6+^ were involved in the ICEC reaction.

Steps 3 and 4 were designed to understand the role of individual Zn^*δ*+^ or Cr^6+^ in ICEC. When feeding 100 mTorr C_2_H_6_ (without CO_2_) in step 3 (Fig. 3a, b), the signals of CO_2_ and CO_ads_ species disappeared, which indicates that only ethane dehydrogenation could occur. The proportions of Zn^2+^ and Zn^*δ*+^ remain similar to the case of co-feeding C_2_H_6_ and CO_2_ in step 2, indicating the formation of Zn-H^*δ*-^ hydride during the ethane dehydrogenation. Notice that the proportions of Zn^*δ*+^ and Zn^2+^ are comparable between step 1 (UHV) and step 4 (feeding 100 mTorr CO_2_ without ethane), ruling out the likelihood of oxidation of Zn^*δ*+^ to Zn^2+^ by CO_2_ or its derived intermediates.

In the case of Cr 2*p*_3/2_ XPS spectra (Fig. 3c), the oxidation of Cr^3+^ to Cr^6+^ only occurs when CO_2_ was fed (steps 2 and 4). Previous studies have demonstrated the oxidation of Cr^3+^ to Cr^6+^ in Cr-based catalysts suffers from sluggish O abstraction from CO_2_^20,59^. In our cases, the facile reoxidation of Cr^3+^ to Cr^6+^ may be due to the possibility that nearby ${{{{{\rm{Zn}}}}}}^{\delta+}$ facilitate the CO_2_ activation to enable easier O abstraction. The CO_ads_ species at 285.5 eV were detected in the presence/co-presence of CO_2_ in the C 1 s spectra (Fig. 3a, steps 2 and 4), which further demonstrates facile CO_2_ dissociation over the Zn^*δ*+^-O-Cr^6+^ site. Our DFT calculation (Fig. 3e) indicates that the intermediate of CO_2_ activation is carboxyl (COOH*). The decomposition of COOH* requires the participation of the Cr site through the formation of a new Cr-O bond (d(Cr-O) = 2.08 Å), which will maintain its high oxidation state. In summary, the APXPS results show that binuclear Zn^*δ*+^-O-Cr^6+^ sites serve as atomically synergistic sites for ICEC.

### Atomically synergistic mechanism

**Fig. 4:**
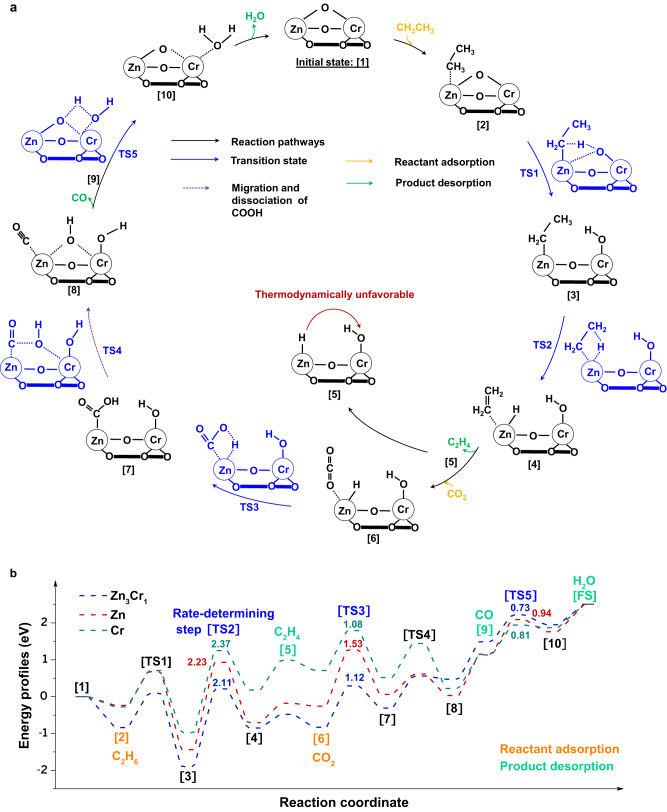
Atomically synergistic mechanism of ICEC over binuclear Zn-O-Cr sites. **a** Catalytic cycles of ICEC over binuclear Zn-O-Cr sites of Zn_3_Cr_1_/SSZ−13. **b** The calculated energy profiles of ICEC on Zn_3_Cr_1_/SSZ−13, Zn/SSZ-13, and Cr/SSZ-13.

We developed atomically synergistic mechanisms for ICEC on binuclear Zn-O-Cr sites (Fig. 4 and Supplementary Fig. 30-35, Supplementary Tables 8-10). Figure 4a shows that the catalytic cycle is initiated by C_2_H_6_ adsorption on Zn ([1] [2]), then heterolytic cleavage of the first C-H bond (0.93 eV) by breaking a Zn-O-Cr bond to form Zn-CH_2_-CH_3_ and Cr-OH ([2] [3]), followed by a homolytic scission of the β-C-H bond (2.11 eV) ([3] [4]), and finally, C_2_H_4_ desorption and Zn-H^*δ*-^ hydride formation ([4] [5]). C_2_H_6_ activation at the Cr of Zn-O-Cr sites has higher energy barriers for the first C-H cleavage (1.78 eV vs 0.93 eV) and β-C-H bond dissociation (>3.0 eV vs 2.11 eV), leading to a kinetically unfavorable pathway (Supplementary Figs. 31 and 32). In the second stage (Fig. 4a), CO_2_ prefers to adsorb at Zn-H^*δ*-^ site (−0.35 eV) than the Cr site (−0.05 eV) through acid-base interaction (Supplementary Fig. 33) ([5][6])^60,61^; CO_2_ adsorption energy (−0.35 eV) at Zn-H^*δ*-^ site is lower than ethane adsorption (−0.17 eV), which will help prevent the C_2_H_6_ cracking reaction (Supplementary Table 5). Then Zn-H^*δ*-^ enables CO_2_ hydrogenation ([6][7]) to carboxyl (COOH*), followed by CO-OH cleavage and migration of -OH to be shared with Cr ([7][8]). Finally, after CO desorption from the Zn site and the formation and desorption of H_2_O from the Cr site to replenish lattice oxygen ([8][10]), the ICEC catalytic cycle is closed.

The simulated catalytic cycles of Zn/SSZ-13 and Cr/SSZ-13 are shown in Supplementary Figs. 34 and 35, and the calculated energy profiles of ICEC over Zn_3_Cr_1_/SSZ-13, Zn/SSZ-13, and Cr/SSZ-13 are presented in Fig. 4b. The rate-determining step of ICEC is the homolytic cleavage of the β-C-H bond in ethane activation [TS2] (Fig. 4b, Supplementary Table 9). Zn_3_Cr_1_/SSZ-13 exhibited a lower activation barrier (2.11 eV) than Zn/SSZ-13 (2.37 eV) and Cr/SSZ-13 (2.23 eV). Moreover, Zn_3_Cr_1_/SSZ-13 also displayed a much lower energy barrier (1.12 eV, 0.73 eV) for CO_2_ activation [TS3] and H_2_O formation [TS5] than Zn/SSZ-13 (1.53 eV, 0.94 eV). This explained the much higher CO_2_ conversion and utilization of Zn_3_Cr_1_/SSZ-13 catalyst than Zn/SSZ-13 catalyst (Fig. 2b). We also found that Zn_3_Cr_1_/SSZ-13 exhibited the most favorable energetics of reactants adsorption and products desorption which also helps accelerate catalytic reactions (Supplementary Table 10). Therefore, the kinetically and thermodynamically favorable elementary steps in the ICEC catalytic cycle result in the highest catalytic performance of Zn_3_Cr_1_/SSZ-13. Its superior performance in ICEC is due to the atomic synergies between the acidic Zn site and the redox Cr site of ABC.

## Discussion

In this study, we demonstrated the advantages of an atomically-synergistic binuclear-site catalyst (ABC) synthesized by colocalizing Zn and Cr sites on a zeolite SSZ-13 support (ZnCr/SSZ-13) for the iso-stoichiometric co-conversion of ethane and CO_2_ (ICEC) reaction. This ZnCr ABC catalyst exhibited exceptional catalytic performance, with 100% ethylene selectivity and 99.0% ${{{{{\rm{U}}}}}}_{{{{{{\rm{CO}}}}}}_{2}}$ under optimized conditions. The combined results of XAS, AP-XPS, and DFT studies show that the electronic properties and catalytic activity of ZnCr/SSZ-13 can be precisely assigned to unique atomic synergies between neighboring Zn and Cr atoms resulting from colocalization. The redox Cr site facilitates the formation of Zn^*δ*+^, which is the active site for easier adsorption and activation of both ethane and CO_2_. Furthermore, the Cr site accelerates CO_2_ dissociation, due to its redox properties, and facilitates the formation and desorption of H_2_O. This combination thus results in rate matching between ethane dehydrogenation and CO_2_ hydrogenation. Our study highlights the importance of atomic synergy, providing guidance for developing novel catalysts with potential economic and ecological benefits in CO_2_ conversion and olefin production.

## Methods

Materials synthesis. Zn_*x*_Cr_*y*_/SSZ-13 (*x* = 1, *y* = 0; *x/y* = 1/2, 1, 2, 3, and 4; *x* = 0, *y* = 1) catalysts were prepared by the dry-deposition method^62^. The total amount of Zn and Cr in all catalysts was set at 0.8 mmol per 1 g of support materials. In a typical synthesis, the SSZ-13 zeolite support was pretreated in a vacuum at 120 °C for 3 h to remove moisture. The stoichiometric amount of Zinc (II) acetate and/or chromium (III) acetate hydroxide (Zn + Cr = 0.8 mmol) was thoroughly mixed with 1 g of SSZ-13 zeolites using an analog vortex mixer. The resulting solid mixtures were sufficiently ground in glove box for 20 min. Subsequently, the samples are heated in flowing N_2_ (100 mL/min) at 550 °C for 3 h at a ramping rate of 2 °C/min and subsequently in flowing air (100 mL/min) for another 3 h. Both N_2_ and air were purified by moisture trap (Restek). Afterward, the resulting materials were pretreated through Na^+^-neutralization. Typically, the sample was firstly dispersed in deionized water and then dropwise addition of sodium bicarbonate solution (0.01 M, pH = 8) was performed until the solution reached a pH of 7. The slurry was then collected and dried at 110 °C overnight. The samples were finally calcined in static air at 500 °C for 2 h with a ramping rate of 2 °C/min. The final products were stored in an N_2_ box. The control samples of Zn_x_Cr/SSZ-13 (x = 1/2, 1, and 3) were synthesized using the traditional co-precipitation (CP) method according to the reported recipe^41^, followed by Na^+^ neutralization.

Materials characterization. Nitrogen physisorption was performed on the Quantachrome Autosorb iQ2 instrument at 77 K to obtain textural information. The surface area (S_BET_) was determined from the N_2_ isotherms using the Brunauer-Emmett-Teller (BET) method. X-ray fluorescence (XRF) measurements were performed with a EDAX Orbis Micro-XRF Spectrometer. The attenuated total reflection Fourier transform infrared (ATR-FTIR) measurements were conducted by a Thermo Nicolet iS50 FTIR spectrometer with a diamond crystal ATR module. Powder X-ray diffraction (XRD) analysis was performed on a Rigaku MiniFlex 6 G X-Ray Diffractometer (Cu K_α_ radiation with wavelength of 1.5406 Å). The transmission electron microscopy (TEM) experiments were performed on a FEI ThemIS aberration-corrected TEM at the National Center for Electron Microscopy (NCEM) of the Molecular Foundry (MF), Lawrence Berkeley National Laboratory (LBNL). The microscope was operated at 300 keV with a Super-X energy dispersive X-ray spectroscopy (EDS) detector, allowing for rapid chemical identification. X-ray photoelectron spectroscopy (XPS) measurement was conducted at a K-Alpha Plus XPS spectrometer (Thermo Scientific), which consists of a monochromatic Al X-ray source (Al *K*_α_ = 1,486.68 eV) with variable spot size ranging from 30 microns to 400 microns. Powder samples were placed on a double-sided silver tape and the spectra were acquired using the flood-gun source to account for surface charging. All the spectra were analyzed using the CasaXPS software package.

Zn *L*_3_-edge soft X-ray absorption spectroscopy (sXAS) measurement was performed at Beamline 7.3.1 of the Advanced Light Source (ALS) at Lawrence Berkeley National Laboratory (LBNL). sXAS measurement was collected at room temperature through total electron yield (TEY) mode with a probe depth of no more than 10 nm. All the TEY spectra were normalized to the beam flux.

The measurements of Zn *K*-edge and Cr *K*-edge XAS spectra including X-ray absorption near edge structure (XANES) and extended X-ray absorption fine structure (EXAFS) were performed at TPS 44 A beamline in National Synchrotron Radiation Research Center (NSRRC) in Taiwan. The data were collected in fluorescence mode by using 7-element silicon drift detector and the Zn and Cr metal foil were used as references for the energy calibration. The data were processed according to standard procedures using Demeter program package.

Ambient-pressure XPS (AP-XPS) analysis was conducted at Sandia National Laboratories (Livermore, CA) using a differentially-pumped Al Kα source (Specs model XR50) with a photon energy of 1486.6 keV. Emitted photo- (and Auger) electrons were detected using a near-ambient pressure hemispherical analyzer (Specs model Phoibos 150) mounted in a custom designed system capable of measuring XPS under sample gas pressures up to 10 Torr. We used baked steel gas lines and leak valves to introduce C_2_H_6_ (99.995% pure) and CO_2_ (99.999% pure) from Matheson Tri-Gas Inc. XPS/Auger peak locations, widths, and areas were obtained using a Shirley background subtraction and by fitting the data to mixed Gaussian-Lorentzian line shapes using CasaXPS software.

Performance Tests. The catalytic performance of iso-stoichiometric co-conversion of ethane and CO_2_ (ICEC) was conducted on a continuous fix-bed reactor in the temperature range of 400–550 °C and under ambient pressure, which is held inside an electric furnace with temperature controlled by a K-type thermocouple. In a typical catalytic measurement, a total of 200 mg catalyst diluted by 800 mg sand was loaded into the middle of the reactor plugged by quartz wool on two sides. Before the catalytic test, the catalyst bed was pretreated under a flow of Argon (25 sccm) at 550 °C for 1 h with a ramping rate of 10  °C from room temperature. Afterward, the reactant mixtures consisting of 5% CO_2_, and 5% C_2_H_6_ balanced with Argon were introduced with a total flow rate of 25 sccm. Argon is used as an internal standard. The reaction products were analyzed by online GC (Agilent 5890, ShinCarbon ST Packed Columns) equipped with thermal conductivity detector (TCD) and flame ionization detector (FID). To better reveal the reaction kinetics, apparent activation barriers were determined in the temperature range of 400–475 °C (The conversions of CO_2_ and C_2_H_6_ are less than 10%). The carbon and oxygen balances were within 100 ± 2% for all tests. The conversions of CO_2_ and C_2_H_6_, C_2_H_4_ selectivity, utilization of converted CO_2_ (${{{{{\rm{U}}}}}}_{{{{{{\rm{CO}}}}}}_{2}}$), C_2_H_4_ yield, the turnover frequency (TOF) of C_2_H_4_ formation, and the space time yield (STY) of C_2_H_4_ formation were calculated as follows (where n denotes molar flow of substance (mol/min), *n*_*Zn+Cr*_ means the total molar loading of Zn and/or Cr, ${{{{{\rm{M}}}}}}_{{{{{{\rm{C}}}}}}_{2}{{{{{\rm{H}}}}}}_{4}}$ is the molecular weight of C_2_H_4_ (28 g/mol) and m_cat_ stands for catalyst mass (kg)):

1 $${{{{\rm{CO}}}}}_2\, {{{{\rm{conversion}}}}}\, (\%)=\frac{{{{{{\rm{n}}}}}}_{{{{{{\rm{CO}}}}}}_{2}{{{{\rm{input}}}}}}-{{{{{\rm{n}}}}}}_{{{{{{\rm{CO}}}}}}_{2}{{{{\rm{output}}}}}}}{{{{{{\rm{n}}}}}}_{{{{{{\rm{CO}}}}}}_{2}{{{{\rm{input}}}}}}}\times 100\%$$

2 $${{{{\rm{C}}}}}_2{{{{\rm{H}}}}}_6\, {{{{\rm{conversion}}}}}\, (\%)=\frac{{{{{{\rm{n}}}}}}_{{{{{{\rm{C}}}}}}_{2}{{{{{\rm{H}}}}}}_{6}{{{{\rm{input}}}}}}-{{{{{\rm{n}}}}}}_{{{{{{\rm{C}}}}}}_{2}{{{{{\rm{H}}}}}}_{6}{{{{\rm{output}}}}}}}{{{{{{\rm{n}}}}}}_{{{{{{\rm{C}}}}}}_{2}{{{{{\rm{H}}}}}}_{6}{{{{\rm{input}}}}}}}\times 100\%$$

3 $${{{{\rm{C}}}}}_2{{{{\rm{H}}}}}_4\, {{{{\rm{selectivity}}}}}\, (\%)=\frac{{{{{{\rm{n}}}}}}_{{{{{{\rm{C}}}}}}_{2}{{{{{\rm{H}}}}}}_{4}{{{{\rm{output}}}}}}}{{{{{{\rm{n}}}}}}_{{{{{{\rm{C}}}}}}_{2}{{{{{\rm{H}}}}}}_{6}{{{{\rm{input}}}}}}-{{{{{\rm{n}}}}}}_{{{{{{\rm{C}}}}}}_{2}{{{{{\rm{H}}}}}}_{6}{{{{\rm{output}}}}}}}\times 100\%$$

4 $${{{{{\rm{U}}}}}}_{{{{{{\rm{CO}}}}}}_{2}}(\%)=\frac{{{{{{\rm{CO}}}}}}_{2}\, {{{{\rm{conversion}}}}}}{{{{{{\rm{C}}}}}}_{2}{{{{{\rm{H}}}}}}_{6}\, {{{{\rm{conversion}}}}}}\times 100\%$$

5 $${{{{\rm{C}}}}}_2{{{{\rm{H}}}}}_4\, {{{{\rm{yield}}}}}\, (\%)={{{{\rm{C}}}}}_2{{{{\rm{H}}}}}_6\, {{{{\rm{conversion}}}}}\times {{{{\rm{C}}}}}_2{{{{\rm{H}}}}}_4\, {{{{\rm{selectivity}}}}}$$

6 $${{{{\rm{TOF}}}}}=\frac{{{{{{\rm{n}}}}}}_{{{{{{\rm{C}}}}}}_{2}{{{{{\rm{H}}}}}}_{4}{{{{\rm{output}}}}}}\times 60}{{n}_{{Zn}+{Cr}}}$$

7 $${{{{\rm{STY}}}}}=\frac{{{{{{\rm{n}}}}}}_{{{{{{\rm{C}}}}}}_{2}{{{{{\rm{H}}}}}}_{4}{{{{\rm{output}}}}}}\times {{{{{\rm{M}}}}}}_{{{{{{\rm{C}}}}}}_{2}{{{{{\rm{H}}}}}}_{4}}\times 60}{1000\times {{{{{{\rm{m}}}}}}}_{{{{{{\rm{cat}}}}}}}}$$

It is noted that calculations of ${{{{{\rm{U}}}}}}_{{{{{{\rm{CO}}}}}}_{2}}$ were also calibrated and examined by the equation:

8 $${{{{{\rm{U}}}}}}_{{{{{{\rm{CO}}}}}}_{2}}(\%)=\frac{{{{{{\rm{n}}}}}}_{{{{{\rm{CO output}}}}}}}{{{{{{\rm{n}}}}}}_{{{{{{\rm{C}}}}}}_{2}{{{{{\rm{H}}}}}}_{6}{{{{\rm{input}}}}}}-{{{{{\rm{n}}}}}}_{{{{{{\rm{C}}}}}}_{2}{{{{{\rm{H}}}}}}_{6}{{{{\rm{output}}}}}}}\times 100\%$$

Computational Methods. All DFT calculations were performed using Vienna ab initio simulation package (VASP)^63,64^. The projector-augmented wave (PAW) method was used to represent the core-valence electron interaction^65,66^. The generalized gradient approximation (GGA) with the Perdew–Burke–Ernzerhof (PBE) exchange-correlation functional was used with D3 dispersion correction^67,68^. The cutoff energy for the planewave basis was 500 eV. To accurately treat the highly localized transition metal 3*d* orbitals, the spin-polarized DFT + U approach^69,70^ was employed: U_eff_ = 4.7 and 3.0 eV were applied to the Zn 3*d* and Cr 3*d* state, respectively^71,72^. Electronic energies were converged to within 10^−4^ eV and the atomic positions were relaxed until the force on each atom was less than 0.05 eV/Å. The climbing-image nudged elastic band (CI-NEB) method^73^ was used to search for the transition states. A hexagonal unit cell of the SSZ-13 molecular sieve^74^ was used as the support: *a* = *b* = 13.72 Å, *c* = 14.95 Å; composition: Na_1_Al_1_Si_35_O_72_. Only the Γ-point was used to sample the Brillouin zone. The unit cell contains two hexagonal prisms; two units of Zn_2_O_2_ and Cr_2_O_3_ were placed on the two prisms, to create the models of Zn/SSZ-13 and Cr/SSZ-13, respectively; one Zn_2_O_2_ unit was placed on one of the prisms and one ZnCrO_2_ unit on the other, to create the model of Zn_3_Cr_1_/SSZ-13 (see Supplementary Fig. 30).

### Supplementary information

Supplementary Information

Peer Review File

## Data Availability

The data that supports the findings of this study are available from the corresponding authors upon request.

## References

[1] Wang H (2021). Strong metal–support interactions on gold nanoparticle catalysts achieved through Le Chatelier’s principle. Nat. Catal..

[2] Parastaev A (2020). Boosting CO_2_ hydrogenation via size-dependent metal–support interactions in cobalt/ceria-based catalysts. Nat. Catal..

[3] Van Deelen TW, Hernández Mejía C, De Jong KP (2019). Control of metal-support interactions in heterogeneous catalysts to enhance activity and selectivity. Nat. Catal..

[4] Chen A (2019). Structure of the catalytically active copper–ceria interfacial perimeter. Nat. Catal..

[5] Lunkenbein T, Schumann J, Behrens M, Schlögl R, Willinger MG (2015). Formation of a ZnO overlayer in industrial Cu/ZnO/Al_2_O_3_ catalysts induced by strong metal-support interactions. Angew.Chem. Int. Ed..

[6] Pu T, Zhang W, Zhu M (2023). Engineering heterogeneous catalysis with strong metal–support interactions: characterization, theory and manipulation. Angew. Chem. Int. Ed..

[7] Li Y, Zhang Y, Qian K, Huang W (2022). Metal-support interactions in metal/oxide catalysts and oxide-metal interactions in oxide/metal inverse catalysts. ACS Catal..

[8] Zhang L, Zhou M, Wang A, Zhang T (2020). Selective hydrogenation over supported metal catalysts: from nanoparticles to single atoms. Chem. Rev..

[9] Shi J (2013). On the synergetic catalytic effect in heterogeneous nanocomposite catalysts. Chem. Rev..

[10] Ro I (2022). Bifunctional hydroformylation on heterogeneous Rh-WO_x_ pair site catalysts. Nature.

[11] Gao R (2021). Pt/Fe_2_O_3_ with Pt–Fe pair sites as a catalyst for oxygen reduction with ultralow Pt loading. Nat. Energy.

[12] Liu C (2022). Catalytic activity enhancement on alcohol dehydrogenation via directing reaction pathways from single- to double-atom catalysis. J. Am. Chem. Soc..

[13] Yang J (2022). Modulating the strong metal-support interaction of single-atom catalysts via vicinal structure decoration. Nat. Commun..

[14] Li WH, Yang J, Wang D (2022). Long-range interactions in diatomic catalysts boosting electrocatalysis. Angew. Chem. Int. Ed..

[15] Pan Y, Zhang C, Liu Z, Chen C, Li Y (2020). Structural regulation with atomic-level precision: from single-atomic site to diatomic and atomic interface. Catal. Matter.

[16] Zeng Z (2021). Orbital coupling of hetero-diatomic nickel-iron site for bifunctional electrocatalysis of CO_2_ reduction and oxygen evolution. Nat. Commun..

[17] Zheng X (2022). Ru–Co pair sites catalyst boosts the energetics for the oxygen evolution reaction. Angew. Chem. Int. Ed..

[18] Guan E (2020). Supported metal pair-site catalysts. ACS Catal..

[19] Mitchell S, Pérez-Ramírez J (2021). Atomically precise control in the design of low-nuclearity supported metal catalysts. Nat. Rev. Mater..

[20] Gomez E, Yan B, Kattel S, Chen JG (2019). Carbon dioxide reduction in tandem with light-alkane dehydrogenation. Nat. Rev. Chem..

[21] Biswas AN, Xie Z, Chen JG (2022). Can CO_2_-assisted alkane dehydrogenation lead to negative CO_2_ emissions?. Joule.

[22] Myint MNZ, Yan B, Wan J, Zhao S, Chen JG (2016). Reforming and oxidative dehydrogenation of ethane with CO_2_ as a soft oxidant over bimetallic catalysts. J. Catal..

[23] Xie Z (2020). Reactions of CO_2_ and ethane enable CO bond insertion for production of C3 oxygenates. Nat. Commun..

[24] Chatani Y, Takizawa T, Murahashi S, Sakata Y, Nishimura Y (1961). Crystal structure of polyketone (1: 1 Ethylene/Carbon Monoxide Copolymer). J. Polym. Sci..

[25] Brubaker MM, Coffman DD, Hoehn HH (1952). Synthesis and characterization of ethylene/carbon monoxide copolymers, a new class of polyketones. J. Am. Chem. Soc..

[26] Chen S (2022). Cationic P,O‐coordinated Nickel(II) catalysts for carbonylative polymerization of ethylene: unexpected productivity via subtle electronic variation. Angew. Chem. Int. Ed..

[27] Ortmann P, Wimmer FP, Mecking S (2015). Long-spaced polyketones from ADMET copolymerizations as ideal models for ethylene/CO copolymers. ACS Macro Lett..

[28] Xie Z, Wang X, Chen X, Liu P, Chen JG (2022). General descriptors for CO_2_-assisted selective C-H/C-C bond scission in ethane. J. Am. Chem. Soc..

[29] Kattel S, Chen JG, Liu P (2018). Mechanistic study of dry reforming of ethane by CO_2_ on a bimetallic PtNi(111) model surface. Catal. Sci. Technol..

[30] Guo H, Xie Z, Wang X, Chen JG, Liu P (2023). Descriptor-based identification of bimetallic-derived catalysts for selective activation of ethane with CO_2_. EES Catal..

[31] Li G, Liu C, Cui X, Yang Y, Shi F (2021). Oxidative dehydrogenation of light alkanes with carbon dioxide. Green Chem..

[32] Najari S (2021). Oxidative dehydrogenation of ethane: catalytic and mechanistic aspects and future trends. Chem. Soc. Rev..

[33] Zhao D (2022). Controlling reaction-induced loss of active sites in ZnO_x_/Silicalite-1 for durable nonoxidative propane dehydrogenation. ACS Catal..

[34] Schweitzer NM (2014). Propylene hydrogenation and propane dehydrogenation by a single-site Zn^2+^ on silica catalyst. ACS Catal..

[35] Zhao D (2021). In situ formation of ZnO_x_ species for efficient propane dehydrogenation. Nature.

[36] Liu X (2022). In situ spectroscopic characterization and theoretical calculations identify partially reduced ZnO_1-x_/Cu interfaces for methanol synthesis from CO_2_. Angew. Chem. Int. Ed..

[37] Consonni M, Jokic D, Murzin DY, Touroude R (1999). High performances of Pt/ZnO catalysts in selective hydrogenation of crotonaldehyde. J. Catal..

[38] Hsu JY (2022). Probing local structural changes by sharp luminescent infrared nanophosphor for application in light-emitting diodes. Chem. Mater..

[39] Liu BM (2023). A High-efficiency blue-LED-excitable NIR-II-emitting MgO:Cr^3+^,Ni^2+^ phosphor for future broadband light source toward multifunctional NIR spectroscopy applications. Chem. Eng. J..

[40] Jiao F (2016). Selective conversion of syngas to light olefins. Science..

[41] Yang F (2019). One-step alkylation of benzene with Syngas over non-noble catalysts mixed with modified HZSM-5. Ind. Eng. Chem. Res..

[42] Song H (2017). Spinel-structured ZnCr_2_O_4_ with excess Zn is the active ZnO/Cr_2_O_3_ catalyst for high-temperature methanol synthesis. ACS Catal..

[43] Li S (2021). Low-valence Zn^δ+^ (0<δ<2) single-atom material as highly efficient electrocatalyst for CO_2_ reduction. Angew. Chem. Int. Ed..

[44] Li X (2019). Improved catalytic performance of ethane dehydrogenation in the presence of CO_2_ over Zr-promoted Cr/SiO_2_. ACS Omega.

[45] Dadlani A (2017). Revealing the bonding environment of Zn in ALD Zn(O,S) buffer layers through X-ray absorption spectroscopy. ACS Appl. Mater. Interfaces.

[46] Qiao L (2013). The impact of crystal symmetry on the electronic structure and functional properties of complex lanthanum chromium oxides. J. Mater. Chem. C.

[47] Ramirez A (2021). Multifunctional catalyst combination for the direct conversion of CO_2_ to propane. JACS Au.

[48] Pinilla-Herrero I (2018). High Zn/Al ratios enhance dehydrogenation vs hydrogen transfer reactions of Zn-ZSM-5 catalytic systems in methanol conversion to aromatics. J. Catal..

[49] Thirumala Bai P (2017). Oxidative dehydrogenation of ethane with carbon dioxide over Cr_2_O_3_/SBA-15 catalysts: the influence of sulfate modification of the support. Appl. Petrochem. Res..

[50] Talati A, Haghighi M, Rahmani F (2016). Oxidative dehydrogenation of ethane to ethylene by carbon dioxide over Cr/TiO_2_–ZrO_2_ nanocatalyst: effect of active phase and support composition on catalytic properties and performance. Adv. Powder Technol..

[51] Shen Z (2009). Dehydrogenation of ethane to ethylene over a highly efficient Ga_2_O_3_/HZSM-5 catalyst in the presence of CO_2_. Appl. Catal. A Gene..

[52] Bugrova TA (2019). Oxidative dehydrogenation of ethane with CO_2_ over CrO_x_ catalysts supported on Al_2_O_3_, ZrO_2_, CeO_2_ and Ce_x_Zr_1-x_O_2_. Catal. Today.

[53] Koirala R, Buechel R, Krumeich F, Pratsinis SE, Baiker A (2015). Oxidative dehydrogenation of ethane with CO_2_ over flame-made Ga-loaded TiO_2_. ACS Catal..

[54] Liu J (2022). Influence of the zeolite surface properties and potassium modification on the Zn-catalyzed CO_2_-assisted oxidative dehydrogenation of ethane. Appl. Catal. B Environ..

[55] Liu J (2021). Highly-dispersed zinc species on zeolites for the continuous and selective dehydrogenation of ethane with CO_2_ as a soft oxidant. ACS Catal..

[56] Tu C (2022). CO_2_-assisted ethane aromatization over zinc and phosphorous modified ZSM-5 catalysts. Appl. Catal. B Environ..

[57] Miller AV, Kaichev VV, Prosvirin IP, Bukhtiyarov VI (2013). Mechanistic study of methanol decomposition and oxidation on Pt(111). J. Phys. Chem. C.

[58] Wu CH, Eren B, Bluhm H, Salmeron MB (2017). Ambient-pressure X-ray photoelectron spectroscopy study of cobalt foil model catalyst under CO, H_2_, and their mixtures. ACS Catal..

[59] Mimura N, Takahara I, Inaba M, Okamoto M, Murata K (2002). High-performance Cr/H-ZSM-5 catalysts for oxidative dehydrogenation of ethane to ethylene with CO_2_ as an oxidant. Catal. Commun..

[60] Álvarez A (2017). CO_2_ activation over catalytic surfaces. ChemPhysChem.

[61] Zimmerman PM, Zhang Z, Musgrave CB (2010). Simultaneous two-hydrogen transfer as a mechanism for efficient CO_2_ reduction. Inorg. Chem..

[62] Peeters E (2021). Highly dispersed Sn-beta zeolites as active catalysts for Baeyer-Villiger oxidation: the role of mobile, in situ Sn(II)O species in solid-state stannation. ACS Catal..

[63] Kresse G, Furthmüller J (1996). Efficiency of ab-initio total energy calculations for metals and semiconductors using a plane-wave basis set. Comput. Mater. Sci..

[64] Kresse G, Furthmüller J (1996). Efficient iterative schemes for ab initio total-energy calculations using a plane-wave basis set. Phys. Rev. B.

[65] Blöchl PE, Jepsen O, Andersen OK (1994). Improved tetrahedron method far Brilleuin-zane integrations. Phys. Rev. B.

[66] Kresse G, Joubert D (1999). From ultrasoft pseudopotentials to the projector augmented-wave method. Phys. Rev. B.

[67] Grimme S, Antony J, Ehrlich S, Krieg H (2010). A consistent and accurate ab initio parametrization of density functional dispersion correction (DFT-D) for the 94 elements H-Pu. J. Chem. Phys..

[68] Perdew JP, Burke K, Ernzerhof M (1996). Generalized gradient approximation made simple. Phys. Rev. Lett..

[69] Anisimov VI, Aryasetiawan F, Lichtenstein AI (1997). First-principles calculations of the electronic structure and spectra of strongly correlated systems: the LDA + U method. J. Phys. Condens. Matter.

[70] Anisimov VI, Zaanen J, Andersen OK (1991). Band theory and Mott insulators: Hubbard U instead of Stoner I. Phys. Rev. B.

[71] Jin J, Chen J, Wang H, Hu P (2019). Insight into room-temperature catalytic oxidation of NO by CrO_2_(110): A DFT study. Chin. Chem. Lett..

[72] Janotti A, Van De Walle CG (2007). Native point defects in ZnO. Phys. Rev. B.

[73] Henkelman G, Uberuaga BP, Jónsson H (2000). A climbing image nudged elastic band method for finding saddle points and minimum energy paths. J. Chem. Phys..

[74] Zhang R (2014). NO chemisorption on Cu/SSZ-13: a comparative study from infrared spectroscopy and DFT calculations. ACS Catal..

