# Supplementary material for: Atomically synergistic Zn-Cr catalyst for iso-stoichiometric co-conversion of ethane and CO2 to ethylene and CO
Source: Nat Commun. 2024 Jan 30;15:911. doi: 10.1038/s41467-024-44918-8 (PMC10828418; doi:10.1038/s41467-024-44918-8)
Supplement: Supplementary file 1 — Supplementary Information [file 41467_2024_44918_MOESM1_ESM.pdf]

Supplementary Information

Atomically-Synergistic Zn-Cr Catalyst for Iso-Stoichiometric Co-Conversion of Ethane and CO_2 to Ethylene and CO

Ji Yang[1,2†], Lu Wang[3†], Jiawei Wan[4†], Farid El Gabaly[5†], Andre L. Fernandes Cauduro[5], Bernice E. Mills[5], Jeng-Lung Chen[6], Liang-Ching Hsu[7], Daewon Lee[4], Xiao Zhao[4], Haimei Zheng[4], Miquel Salmeron[4], Caiqi Wang[8], Zhun Dong[8], Hongfei Lin[8], Gabor A. Somorjai[9], Fabian Rosner[10], Hanna Breunig[10], David Prendergast[2], De-en Jiang[3,11]*, Seema Singh[5]*, Ji Su[1,2]*

[1]Energy Storage and Distributed Resources Division, Lawrence Berkeley National Laboratory, Berkeley, California 94720, United States

[2]The Molecular Foundry, Lawrence Berkeley National Laboratory, Berkeley, California 94720, United States

[3]Department of Chemistry, University of California, Riverside, California 92521, United States

[4]Materials Science Division, Lawrence Berkeley National Laboratory, Berkeley, California 94720, United States

[5]Sandia National Laboratories, Livermore, California 94551, United States

[6]National Synchrotron Radiation Research Center, Science-Based Industrial Park, Hsinchu 30076, Taiwan

[7]Department of Soil and Environmental Sciences, National Chung Hsing University, Taichung 40227, Taiwan

[8]Gene and Linda Voiland School of Chemical Engineering and Bioengineering, Washington State University, Pullman, WA 99163, United States

[9]Department of Chemistry, University of California, Berkeley, California 94720, United States

[10]Energy Analysis and Environmental Impacts Division, Lawrence Berkeley National Laboratory, Berkeley, California 94720, United States

[11]Department of Chemical and Biomolecular Engineering, Vanderbilt University, Nashville, Tennessee 37235, United States

†J. Y., L. W., J. W., and F. E. G. contributed equally to this work.

*Email: jisu@lbl.gov (J. S.); seema.rose.singh@gmail.com (S. S.); djiang@ucr.edu (D. J.)

Supplementary Methods

Materials

Zeolite (H^+)SSZ-13 with SiO_2/Al_2O_3 ratio of 30 was purchased from ACS Material. Zinc (II) acetate (>99.9%) and chromium (III) acetate hydroxide, sodium bicarbonate (>99.9%), zinc (II) nitrate hexahydrate, chromium(III) nitrate nonahydrate, and ammonium carbonate were purchased from Sigma Aldrich. All the chemicals were used as received without any further purification.

Performance Tests

The performance of all catalyst samples in Fig. 2a and Supplementary Fig. 19 were collected in the initial states under 550 ^{o}C. The stability test was conducted under reaction temperature of 500 ^{o}C, with the highest U_{CO_2} and C_2H_4 selectivity at the decent reactant conversions, over the Zn_3Cr_1/SSZ-13 catalyst.

Thermodynamic analysis

Thermodynamic equilibrium calculations were carried out in Aspen Plus V14.

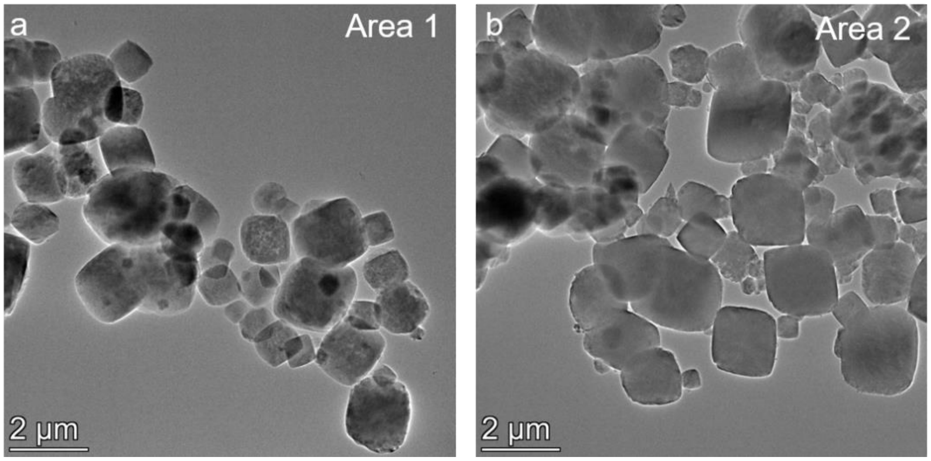

Supplementary Fig. 1 Transmission electron microscopy (TEM) images of different areas of (H^+)SSZ-13 zeolite support: **a**, Area 1; **b**, Area 2.

TEM results suggest that (H^+)SSZ-13 shows cubic morphology with an average crystallite size in the ~1-2 μm range

Supplementary Table 1. Physical characteristics of parent zeolite and as-synthesized catalyst samples.

Catalyst[a]	S_{BET}[b] (m^2 g^{-1})	S_{micro}[c] (m^2 g^{-1})	V_{pore} (cm^3 g^{-1})
(H^+) SSZ-13	488	463	0.253
(Na^+) SSZ-13	445	432	0.223
Cr/SSZ-13	504	482	0.267
Zn_1Cr_2/SSZ-13	474	451	0.247
Zn_1Cr_1/SSZ-13	486	459	0.256
Zn_2Cr_1/SSZ-13	497	475	0.260
Zn_3Cr_1/SSZ-13	499	472	0.263
Zn_4Cr_1/SSZ-13	496	473	0.260
Zn/SSZ-13	498	477	0.260

[a]SSZ-13 support with SiO_2/Al_2O_3 molar ratio of 30 purchased from ACS Material Inc. [b]Brunauer-Emmett-Teller (BET) method applied to the N_2 isotherm. [c]t-plot method applied to the N_2 isotherm

The surface area and pore volume of the parent SSZ-13 zeolite and catalyst samples are comparable, which indicates that the deposition of Zn and Cr species has little influence on pore structure of zeolites.

Supplementary Table 2. Bulk and surface chemical compositions of Zn and Cr in as-synthesized catalysts determined by X-ray fluorescence (XRF) and X-ray photoelectron spectroscopy (XPS), respectively.

	Atomic ratio[a]			
Sample	Zn	Cr	Al	Si
SSZ-13	-	-	3.5	96.5
Zn/SSZ-13	8.7	-	3.7	87.7
Zn_4Cr_1/SSZ-13	7.5	1.8	3.8	86.9
Zn_3Cr_1/SSZ-13	6.6	2.0	4.1	87.3
Zn_1Cr_1/SSZ-13	4.9	3.7	3.8	87.6
Cr/SSZ-13	-	8.6	3.5	87.9

[a]Determined by XRF

	Zn_1Cr_1/SSZ-13	Zn_3Cr_1/SSZ-13	Zn_4Cr_1/SSZ-13
Surface Zn/Cr atomic ratio[a]	1.2	3.2	3.8

[a]Determined by XPS

XRF and XPS results show that surface Zn/Cr ratios is basically consistent with bulk Zn/Cr stoichiometry for Zn_xCr_y catalyst samples (x/y= 4/1, 3/1, and 1).

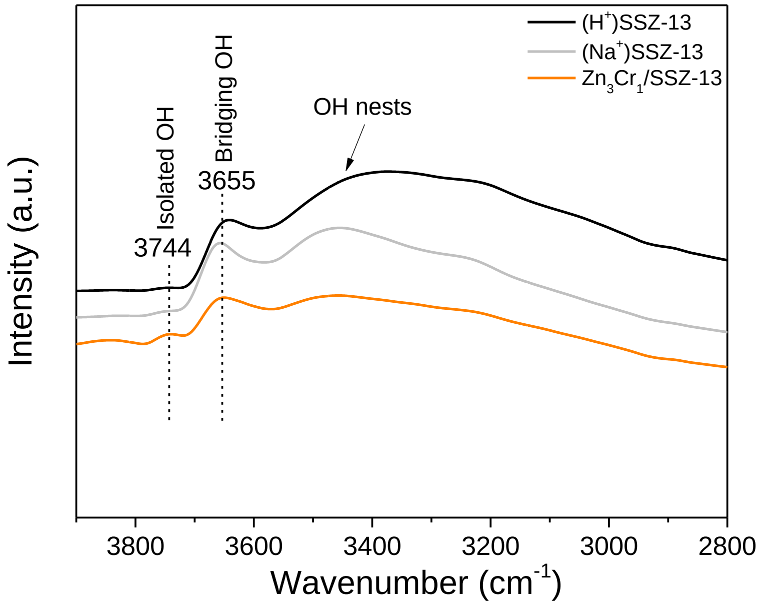

Supplementary Fig. 2. ATR-FTIR spectra of (H^+)SSZ-13, (Na^+)SSZ-13 and as-synthesized ZnCr catalysts.

The bands at 3744, 3655, and ~3427 cm^{-1} correspond to isolated hydroxyl group (OH), bridging OH, and OH nests, respectively[1,2]. After Na^+ modification on (H^+)SSZ-13, the peak intensity of OH nests significantly decreased, indicating the weakened Brønsted acidity. Moreover, the deposition of ZnCrOx species on Zn_3Cr_1/SSZ-13 further reduces the peak intensity of bridging OH and OH nests, indicating a further reduced surface Brønsted acidity.

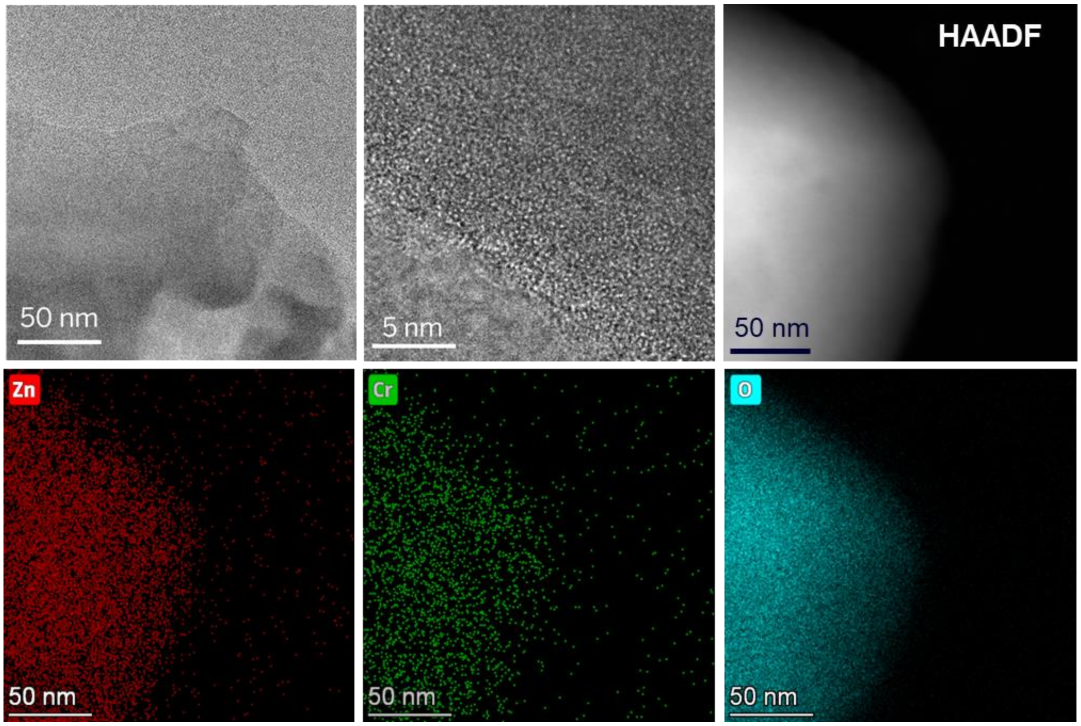

Supplementary Fig. 3. TEM, HRTEM, and HAADF-STEM images of Zn_3Cr_1/SSZ-13 and its elemental maps of Zn, Cr, and O.

No visible nanoparticles are observed in TEM, STEM and elemental mapping results; and no noticeable lattice fringes are tracked in HRTEM.

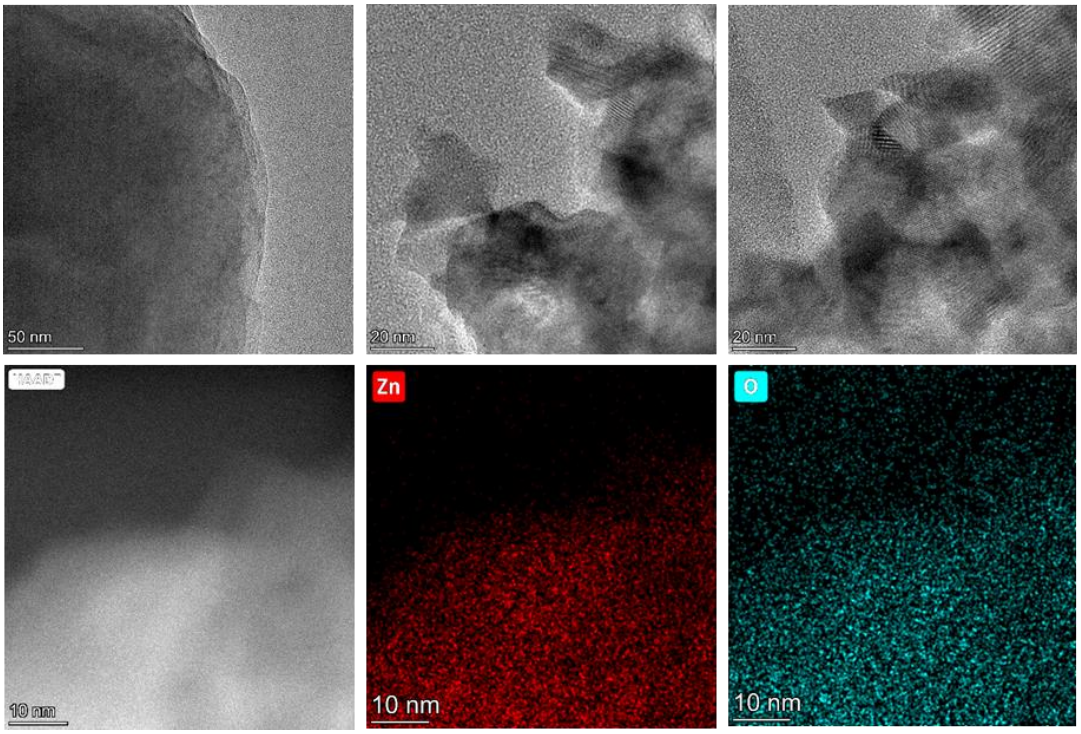

Supplementary Fig. 4. TEM and HAADF-STEM images of Zn/SSZ-13 and its elemental maps of Zn and O.

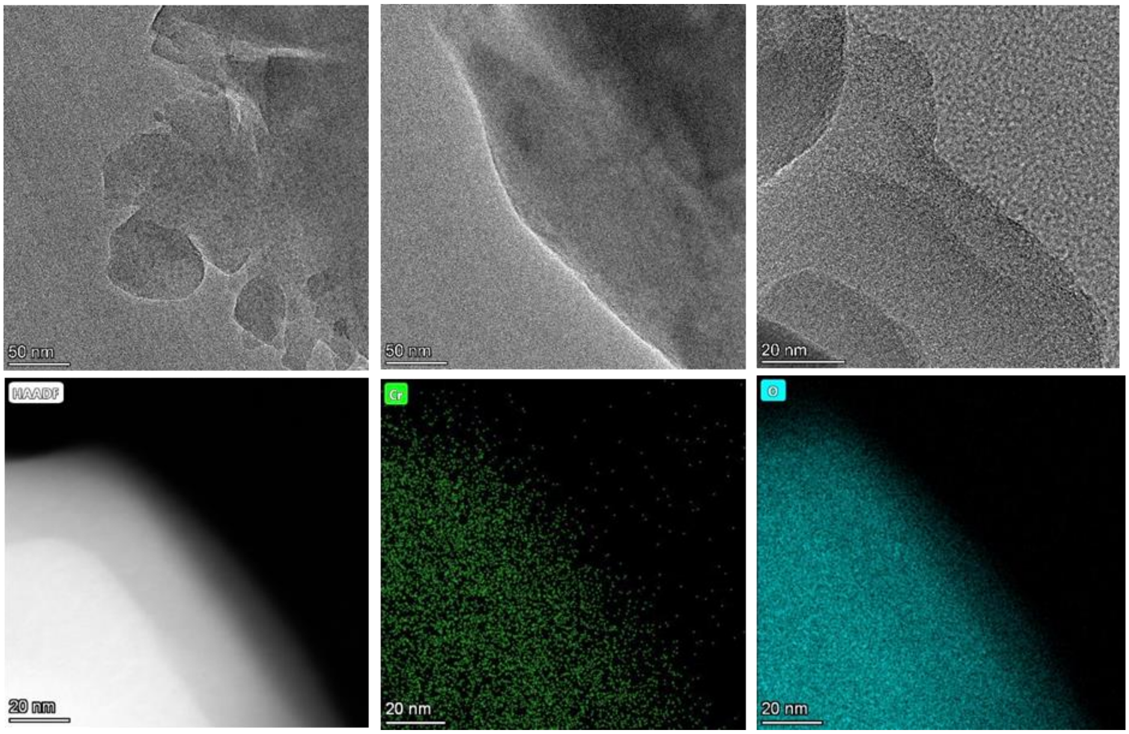

Supplementary Fig. 5. TEM and HAADF-STEM images of Cr/SSZ-13 and its elemental maps of Cr and O.

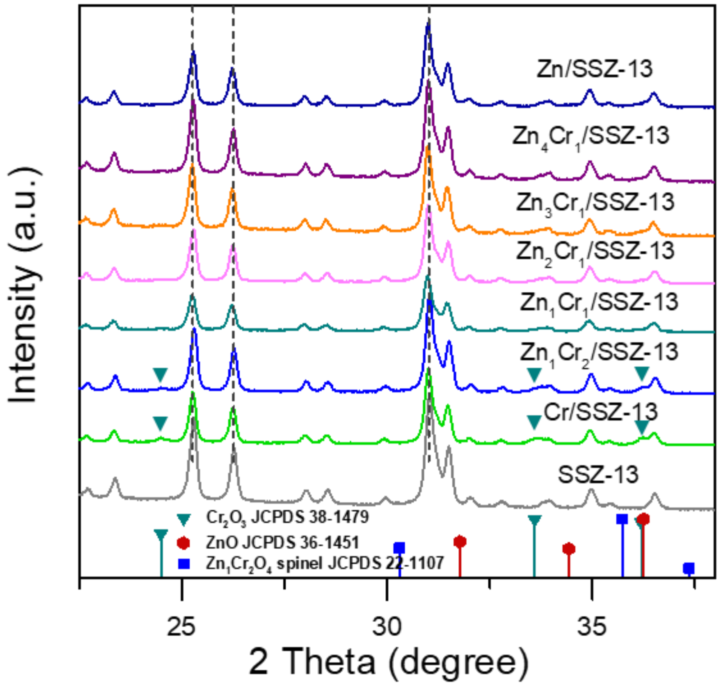

Supplementary Fig. 6. Enlarged XRD patterns of as-prepared catalysts (by dry deposition method) and SSZ-13 support.

As presented in Supplementary Fig. 6, the enlarged XRD patterns of all as-prepared samples by dry deposition method display no characteristic peaks of spinel $ZnCr_2O_4$ and zincite ZnO. And only Cr/SSZ-13 and Zn_1Cr_2/SSZ-13 show the existence of Cr_2O_3 with R3c space group (labeled by green triangle).

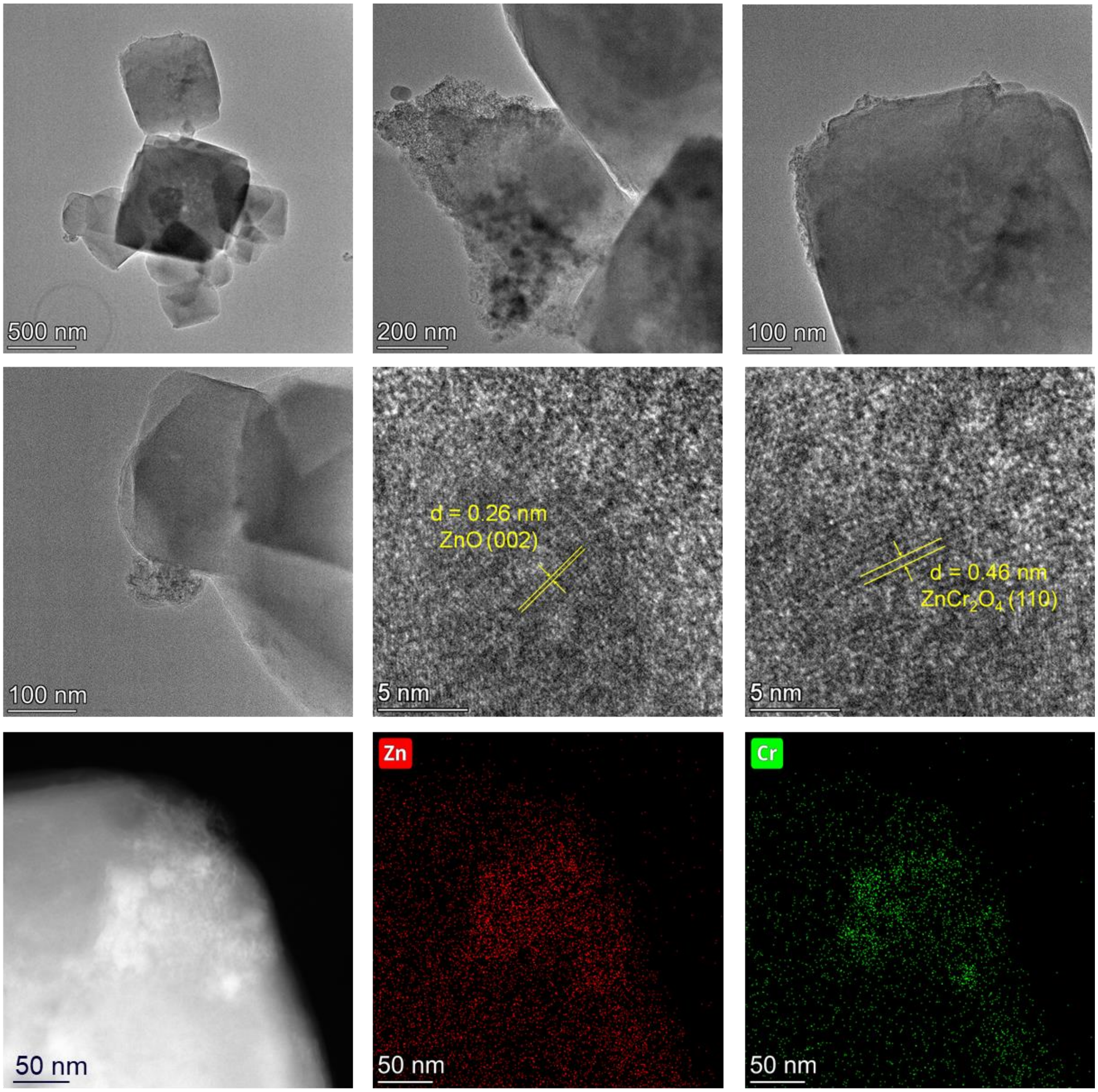

Supplementary Fig. 7. TEM, HRTEM and HAADF-STEM images of Zn_3Cr_1/SSZ-13 (prepared by co-precipitation method) and its elemental maps of Zn and Cr.

TEM images, STEM images and its elemental mapping showed that large $ZnCrO_x$ particles with the size varying from 20 to100 nm are formed for Zn_3Cr_1/SSZ-13 catalysts prepared by co-precipitation (CP) method. Besides, HRTEM analysis indicated the existence of crystallized ZnO and $ZnCr_2O_4$ phase, in consistence of XRD results (see Supplementary Fig. 8 below).

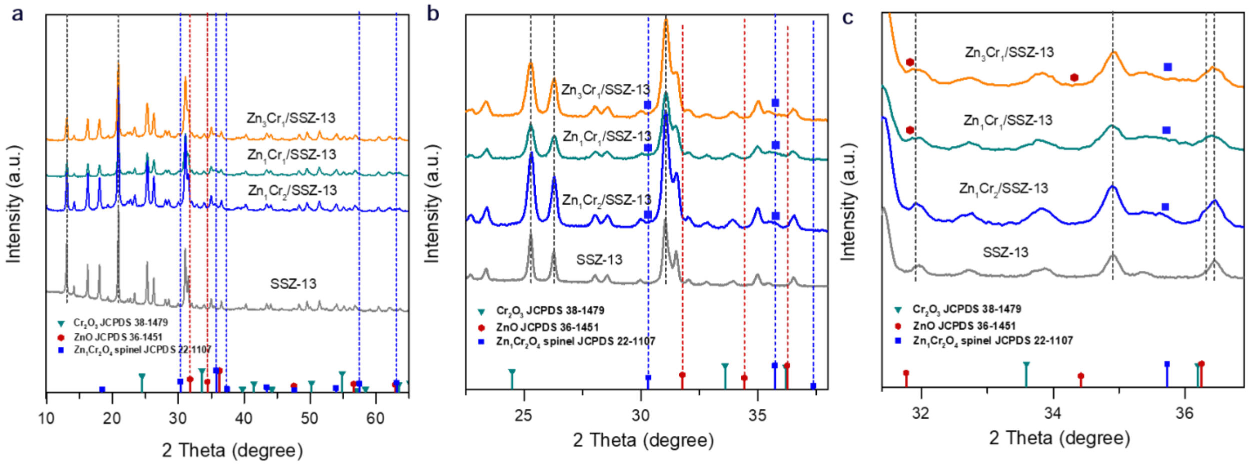

Supplementary Fig. 8. a, X-ray diffraction (XRD) patterns between 10-65°, **b**, partially enlarged XRD patterns between 22.5-38°, **c**, further enlarged XRD patterns between 31.4-36.9° of as-synthesized catalysts (by traditional co-precipitation method) and SSZ-13 support.

For comparison, Zn_1Cr_2/SSZ-13, Zn_1Cr_1/SSZ-13, Zn_3Cr_1/SSZ-13 are synthesized by the traditional co-precipitation (CP) method and their XRD patterns are shown in Supplementary Fig. 8. Phases transitions from spinel $ZnCr_2O_4$ to ZnO are observed as Zn/Cr ratio increased from 1/2 to 3/1. Specifically, sharp reflections of spinel $ZnCr_2O_4$ appeared at Zn/Cr ratio of 1:2. With Zn/Cr ratio increase to 1, spinel $ZnCr_2O_4$ peaks broadens and ZnO reflection sharpens. When Zn/Cr ratio is 3:1, ZnO reflections are dominant and spinel $ZnCr_2O_4$ peaks become weaker.

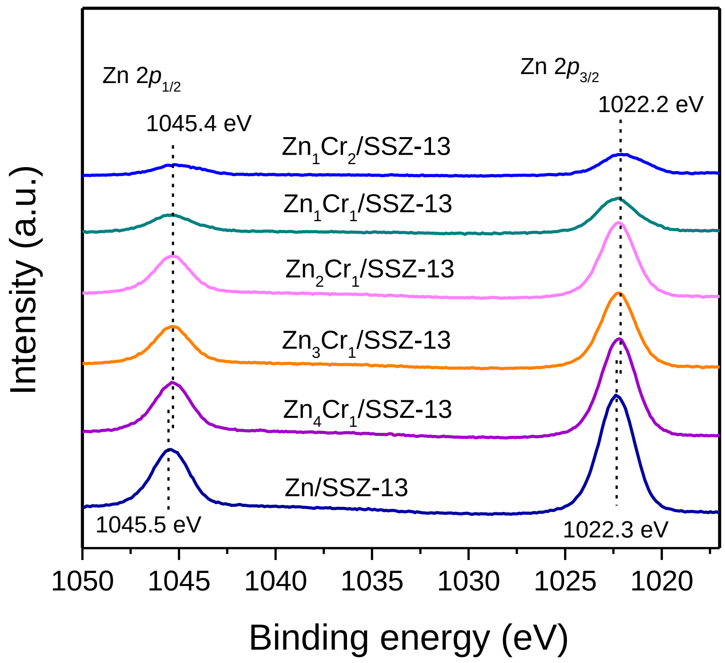

Supplementary Fig. 9. Zn 2*p* XPS spectra for Zn_1Cr_2/SSZ-13, Zn_1Cr_1/SSZ-13, Zn_2Cr_1/SSZ-13, Zn_3Cr_1/SSZ-13, Zn_4Cr_1/SSZ-13, and Zn/SSZ-13.

Compared to Zn/SSZ-13, the binding energy of Zn $2p_{3/2}$ and Zn $2p_{1/2}$ peaks for all Zn-Cr/SSZ-13 samples exhibit a negative shift of 0.1 eV.

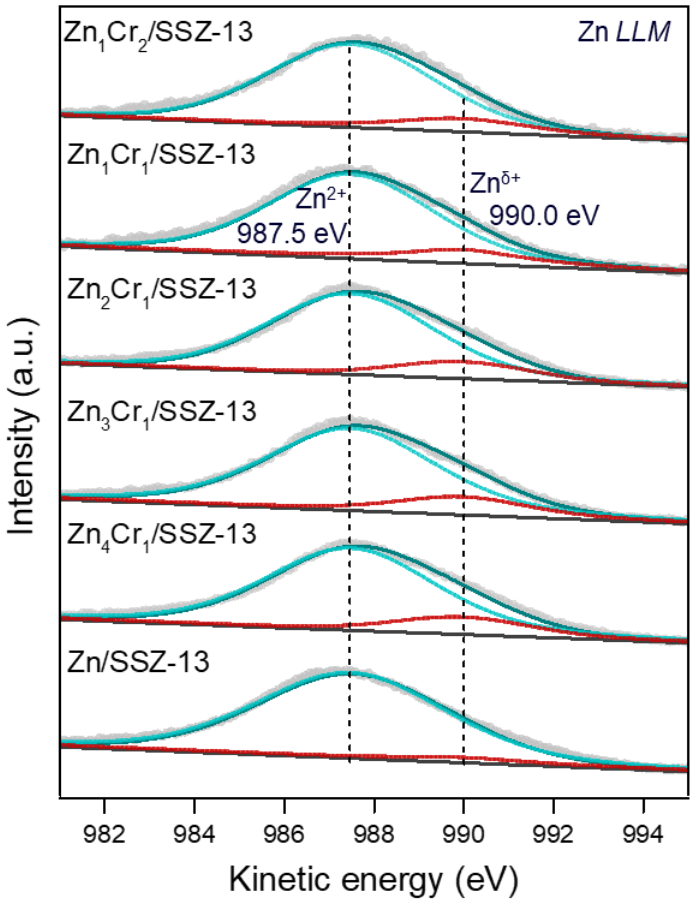

Supplementary Fig. 10. Auger spectra of Zn *LMM* for Zn_1Cr_2/SSZ-13, Zn_1Cr_1/SSZ-13, Zn_2Cr_1/SSZ-13, Zn_3Cr_1/SSZ-13, Zn_4Cr_1/SSZ-13, and Zn/SSZ-13.

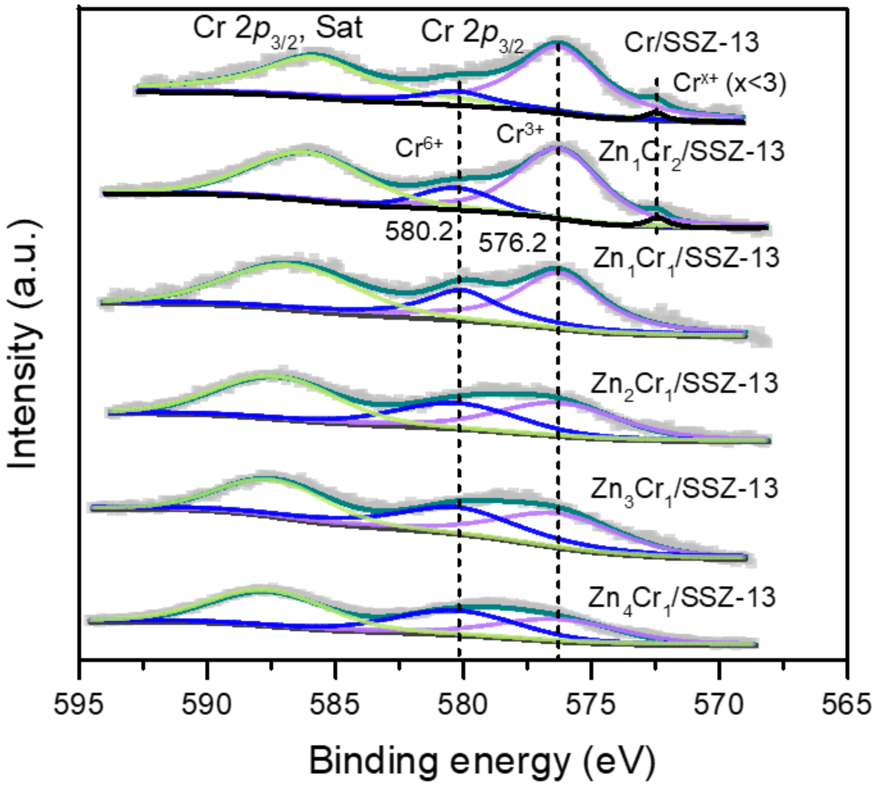

Supplementary Fig. 11. Cr 2$p_{3/2}$ XPS spectra for Cr/SSZ-13, Zn_1Cr_2/SSZ-13, Zn_1Cr_1/SSZ-13, Zn_2Cr_1/SSZ-13, Zn_3Cr_1/SSZ-13, and Zn_4Cr_1/SSZ-13.

Supplementary Table 3. The relative ratio of $Zn^{\delta+}$ and Cr^{6+} in as-synthesized catalysts derived from Auger spectra of Zn *LMM* and Cr $2p_{3/2}$ XPS.

	Cr/SSZ-13	Zn_1Cr_2/ SSZ-13	Zn_1Cr_1/ SSZ-13	Zn_2Cr_1/ SSZ-13	Zn_3Cr_1/ SSZ-13	Zn_4Cr_1/ SSZ-13	Zn/SSZ-13
$Zn^{\delta+}$/ $Zn^{\delta+}+Zn^{2+}$	-	11.2%	12.0%	12.4%	13.4%	11.6%	2.6%
Cr^{6+}/ $Cr^{3+}+Cr^{6+}$	14.0%	17.9%	31.7%	41.1%	49.0%	51.3%	-

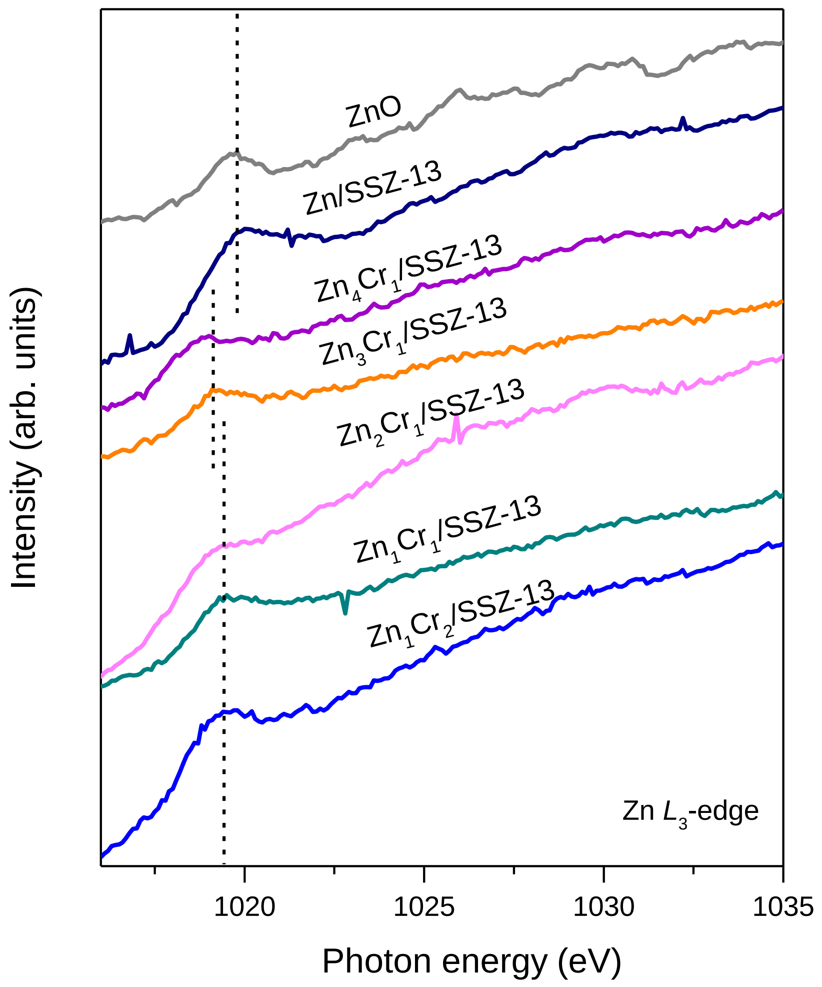

Supplementary Fig. 12. Zn L_3-edge X-ray absorption near-edge structure (XANES) spectra of Zn_1Cr_2/SSZ-13, Zn_1Cr_1/SSZ-13, Zn_2Cr_1/SSZ-13, Zn_3Cr_1/SSZ-13, Zn_4Cr_1/SSZ-13, and Zn/SSZ-13, with ZnO as reference.

The negative energy shifts of Zn 2*p* electrons transition peak indicate decreased oxidation state of Zn.

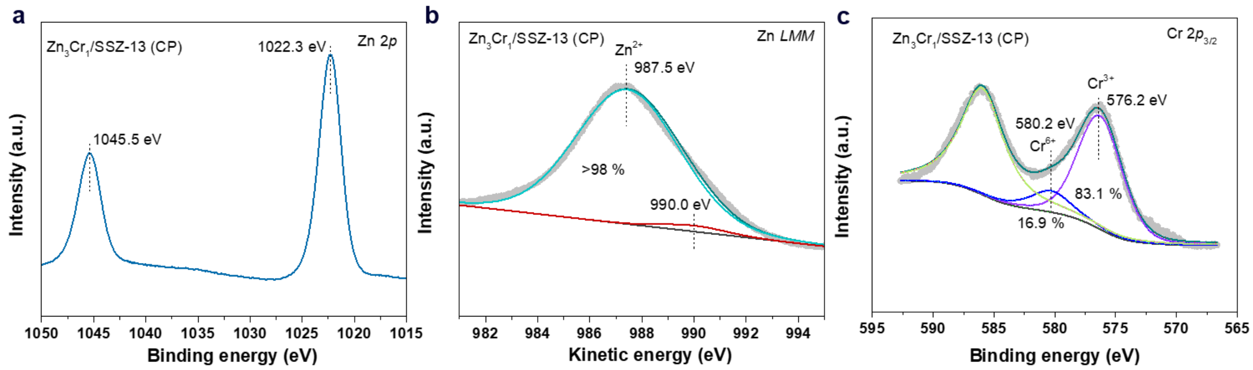

Supplementary Fig. 13. a, Zn 2*p*, **b,** Auger spectra of Zn *LMM*, and **c,** Cr $2p_{3/2}$ XPS spectra for Zn_3Cr_1/SSZ-13 prepared by co-precipitation (CP) method.

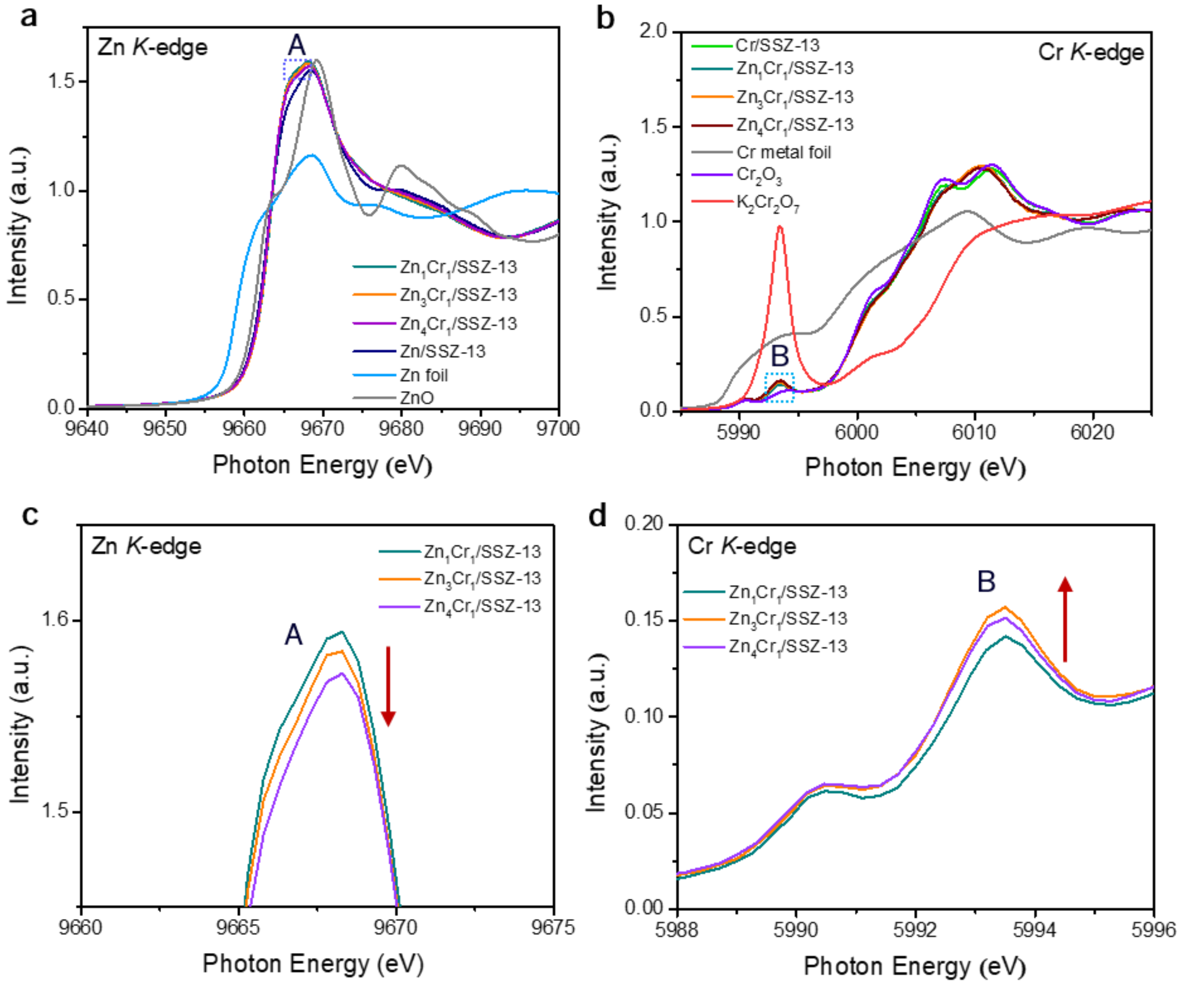

Supplementary Fig. 14. a, Zn *K-edge* X-ray absorption near-edge structure (XANES) spectra of Zn_1Cr_1/SSZ-13, Zn_3Cr_1/SSZ-13, Zn_4Cr_1/SSZ-13, Zn/SSZ-13, with Zn foil and ZnO as references; **b**, Cr *K-edge* XANES spectra of Cr/SSZ-13, Zn_1Cr_1/SSZ-13, Zn_3Cr_1/SSZ-13, Zn_4Cr_1/SSZ-13, with Cr metal foil, Cr_2O_3, and $K_2Cr_2O_7$ as references. **c**, Enlarged electron transition features of Zn 1*s* to Zn 4*p* unoccupied orbitals derived from (**a**); **d**, Enlarged electron transition features of Cr 1*s* to Cr 3*d*-O 2*p* unoccupied orbitals derived from (**b**).

Supplementary Figs. 14a and 14b shows Zn and Cr *K-edge* XANES spectra of as-prepared samples and references. Feature A in Supplementary Fig. 14a demonstrates the electron transition from Zn 1s to Zn 4*p* unoccupied orbitals.[3,4] Feature B in Supplementary Fig. 14b represents electron transition from Cr 1*s* to Cr 3*d*-O 2*p* unoccupied orbitals.[3,5] As Zn/Cr ratio increases from 1/1 to 4/1 in Zn_xCr_y/SSZ-13 catalysts, the intensity of feature A decreases (Supplementary Fig. 14c), suggesting electron occupation in Zn 4*p* unoccupied orbitals; On the contrary, intensity of feature B increases (Supplementary Fig. 14d), which suggests electron transfer out of Cr 3*d*-O 2*p* orbitals near the conduction band minimum. Based on above analyses, we hypothesize there are electronic charge transfers from Cr 3*d*-O 2*p* character in the conduction band to Zn 4*p*, due to a strong Zn-Cr interaction.

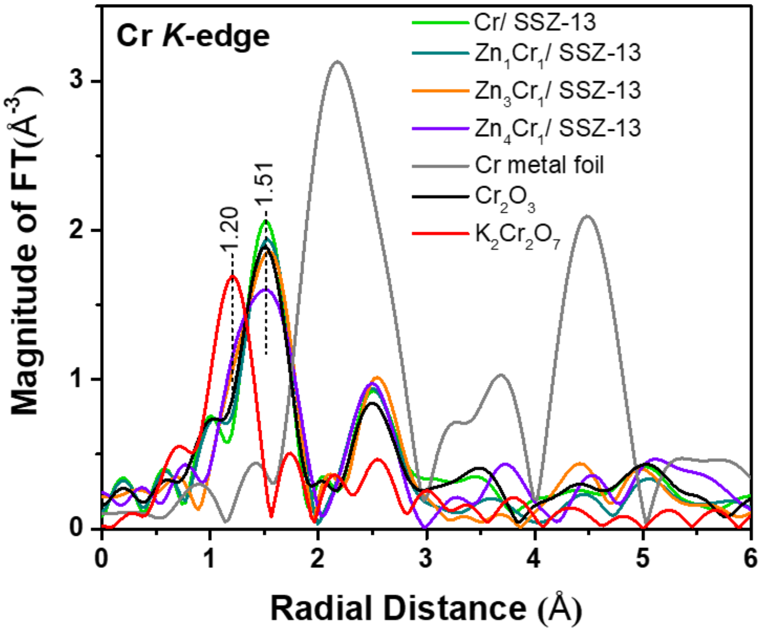

Supplementary Fig. 15. k^2-weighted FT-EXAFS spectra (Cr *K-edge*) of Cr/SSZ-13, Zn_1Cr_1/SSZ-13, Zn_3Cr_1/SSZ-13, and Zn_4Cr_1/SSZ-13, with Cr metal foil, Cr_2O_3, and $K_2Cr_2O_7$ as references.

Supplementary Table 4. EXAFS-derived fitting parameters for Zn-containing samples.

	Path	N	σ^2 (Å^2)	R (Å)	R_f
Zn/SSZ-13	Zn-O	3.96	0.009	1.97	0.012
	Zn-Zn	2.01	0.01	3.22	0.012
Zn_1Cr_1/SSZ-13	Zn-O	4	0.009	1.98	0.003
	Zn-Cr	1.63	0.01	3.44	0.003
Zn_3Cr_1/SSZ-13	Zn-O	3.94	0.008	1.97	0.001
	Zn-Cr	0.86	0.009	3.42	0.001
Zn_4Cr_1/SSZ-13	Zn-O	3.77	0.007	1.97	0.002
	Zn-Cr	1.07	0.013	3.35	0.002

Where N is coordination number, R is distance between absorber and backscatter atoms, σ^2 is Debye–Waller factor value, R_f is R-factor characterizing the goodness of fit. S_o^2 was fixed to 0.84 as determined from ZnO fitting. Error bounds (accuracies) characterizing the structural parameters obtained by EXAFS data analysis are estimated to be as follows: N, ±20%; R, ±1%; and σ^2, ±20%.

Note: Fitting parameters in ZnO reference

ZnO: CN(Zn–O) = 4, r(Zn–O) = 1.970 Å, $CN(Zn–Zn)_I$ = 6, $r(Zn–Zn)_I$ = 3.213 Å, $CN(Zn–Zn)_{II}$ = 6, $r(Zn–Zn)_{II}$ = 3.250 Å.[2]

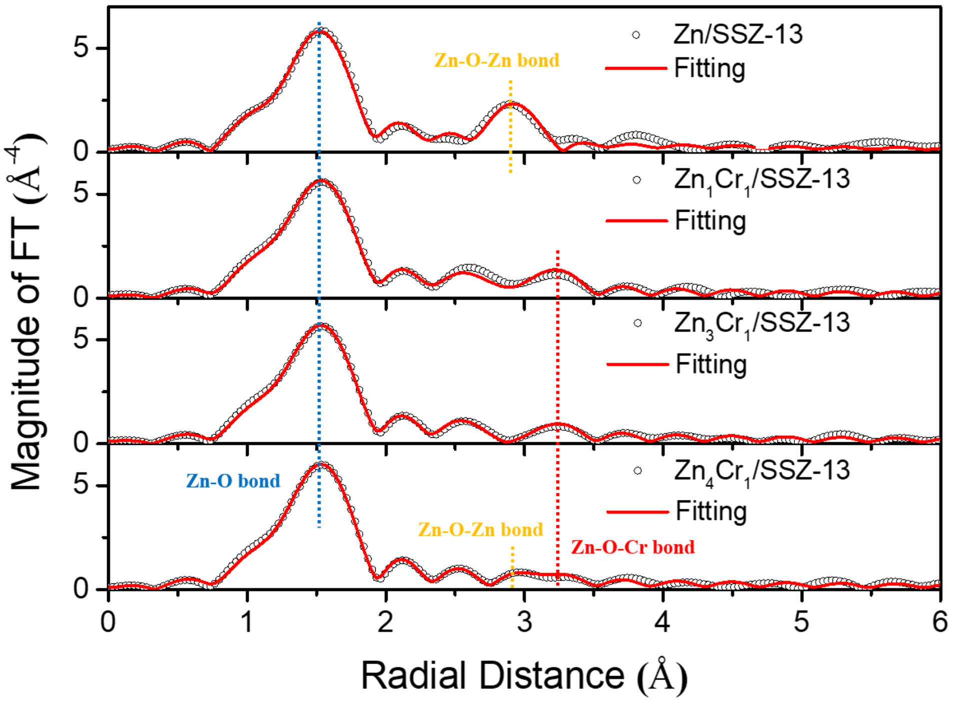

Supplementary Fig. 16. Enlarged k^3-weighted FT-EXAFS spectra and fits (Zn *K-edge*) for Zn/SSZ-13, Zn_1Cr_1/SSZ-13, Zn_3Cr_1/SSZ-13, and Zn_4Cr_1/SSZ-13, with ZnO as reference.

Supplementary Table 5. EXAFS-derived fitting parameters for Cr-containing samples.

	Scattering Path	N	R (Å)	σ^2 (Å^2)	R_f
$K_2Cr_2O_7$	Cr-O	4	1.61	0.004	0.008
Cr_2O_3	Cr-O	6	1.98	0.005	0.01
Cr/SSZ-13	Cr-O	0.7	1.66	0.003	0.002
	Cr-O	6.13	1.96	0.003	0.002
Zn_1Cr_1/SSZ-13	Cr-O	0.31	1.67	0.003	0.007
	Cr-O	5.96	1.98	0.003	0.007
Zn_3Cr_1/SSZ-13	Cr-O	0.71	1.53	0.003	0.007
	Cr-O	5.72	1.99	0.003	0.007
Zn_4Cr_1/SSZ-13	Cr-O	0.49	1.49	0.004	0.012
	Cr-O	5.69	1.98	0.004	0.012

Where N is coordination number, R is distance between absorber and backscatter atoms, σ^2 is Debye–Waller factor value, R_f is R-factor characterizing the goodness of fit. S_o^2 was fixed to 0.72 as determined from Cr_2O_3 fitting. Error bounds (accuracies) characterizing the structural parameters obtained by EXAFS data analysis are estimated to be as follows: N, ±20%; R, ±1%; and σ^2, ±20%.

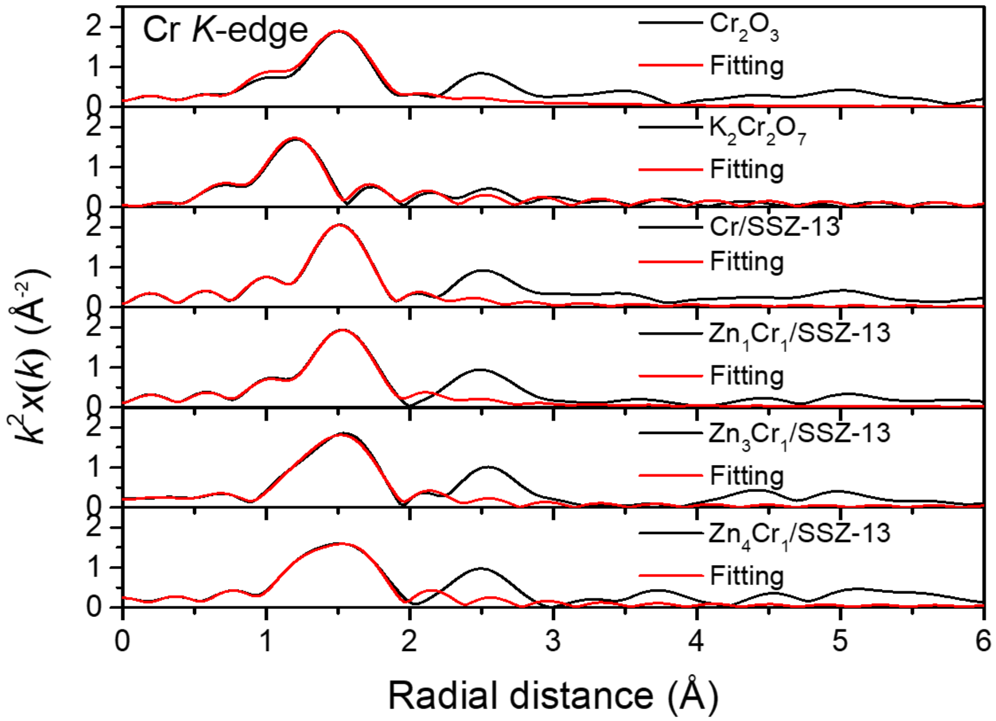

Supplementary Fig. 17. Enlarged k^2-weighted FT-EXAFS spectra and fits (Cr *K-edge*) for Cr/SSZ-13, Zn_1Cr_1/SSZ-13, Zn_3Cr_1/SSZ-13, and Zn_4Cr_1/SSZ-13, with Cr_2O_3 and $K_2Cr_2O_7$ as reference.

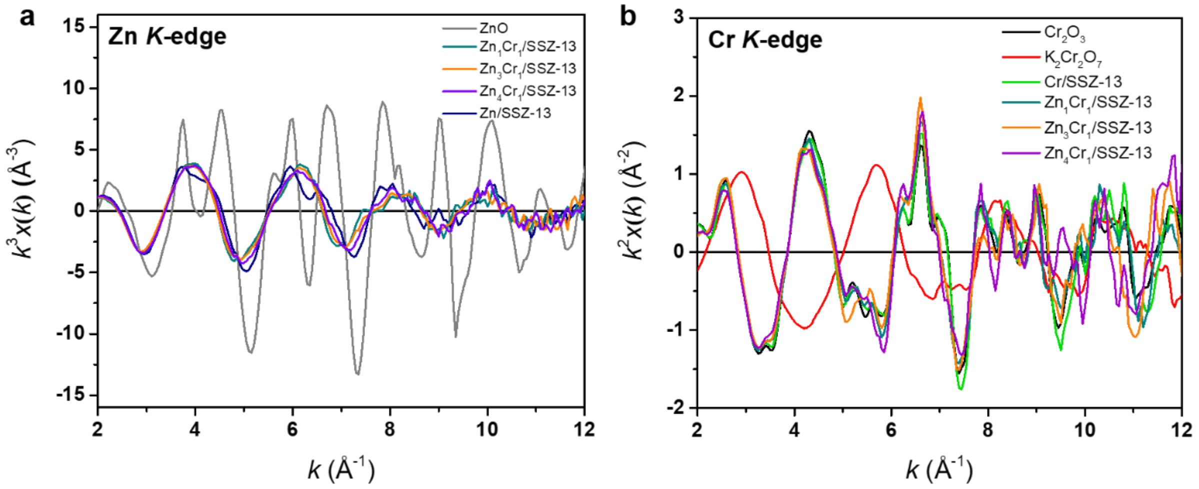

Supplementary Fig. 18. k^3-weighted XAFS spectra for **a**, Zn *K-edge* and k^2-weighted XAFS spectra for **b**, Cr *K-edge* of as-prepared samples and references.

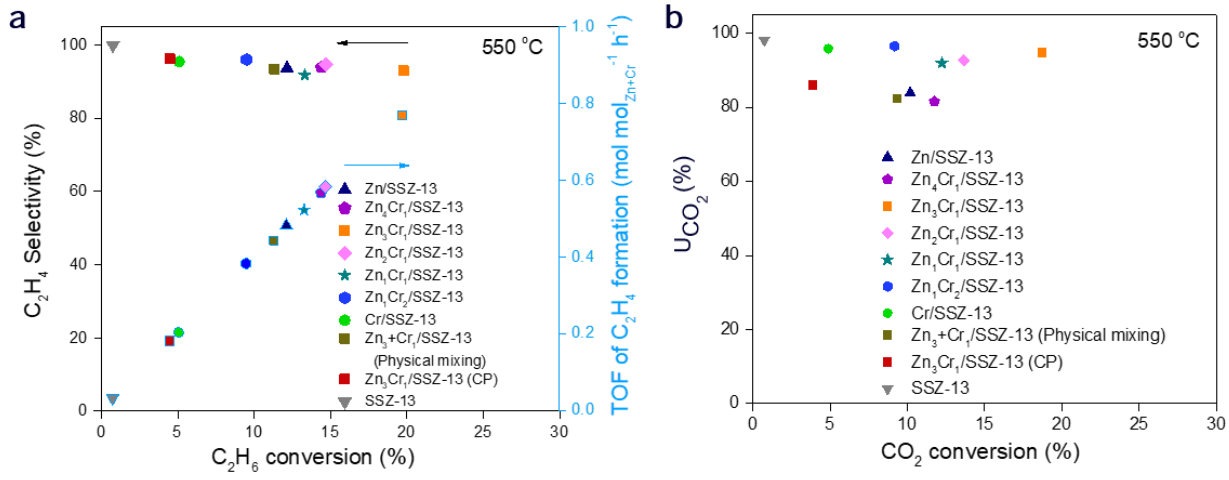

Supplementary Fig. 19. a, C_2H_4 selectivity (left) and turnover frequency (TOF) of C_2H_4 formation (right) as a function of C_2H_6 conversion; **b**, Utilization of converted CO_2 (U_{CO_2}) as a function of CO_2 conversion over as-prepared catalysts.

According to product analysis results, the by-product is methane (CH_4) when C_2H_4 selectivity is less than 100%.

Supplementary Table 6. Catalytic performance comparison for Zn_3Cr_1/SSZ-13 and Cr/SSZ-13 at comparable C_2H_6 and CO_2 conversions.

	Zn_3Cr_1/SSZ-13 (450 ^oC)	Zn_3Cr_1/SSZ-13 (475 ^oC)	Zn_3Cr_1/SSZ-13 (500 ^oC)	Cr/SSZ-13 (550 ^oC)
C_2H_6 Conversion (%)	4.60	6.90	9.60	5.10
CO_2 conversion (%)	4.50	6.80	9.50	4.90
U_{CO_2} (%)	98.0	99.0	99.0	96.0
C_2H_4 selectivity (%)	100	100	100	95.4
TOF of C_2H_4 formation (mol mol_{Zn+Cr}^{-1} h^{-1})	0.19	0.29	0.40	0.20

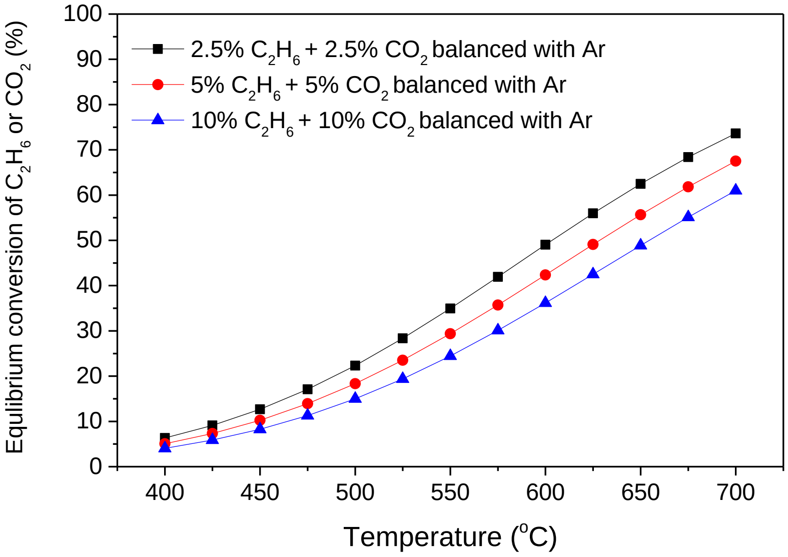

Supplementary Fig. 20. Calculated equilibrium conversions of C_2H_6 and CO_2 for iso-stoichiometric co-conversion of ethane and CO_2 (ICEC) in different concentrations (2.5%, 5%, and 10%).

Supplementary Table 7. Summarization of catalytic behaviors over previously reported catalysts at CO_2/C_2H_6 ratio of 1.

Catalysts	Loading (wt%)	T (°C)	Reactants compositions (C_2H_6 : CO_2 : Ar/He)	WHSV (mL g_{cat}^{-1} h^{-1})	$C_{(C2H6)}$ (%)	$C_{(CO2)}$ (%)	$S_{(C2H4)}$ (%)	$STY_{(C2H4)}$ (kg h^{-1} kg_{cat}^{-1})	U_{CO_2} (%)	Ref.
Zn_3Cr_1/SSZ-13 (oxide)	5	500	1:1:18	7500	9.6	9.5	100	0.045	99.0	This work
Zn_3Cr_1/SSZ-13 (oxide)	5	550	1:1:18	7500	19.8	18.7	93.0	0.086	94.4	This work
$Zn_{9.18}K_{0.74}$/nanoSSZ (oxide)	9.18	550	1:1:18	3600	16.7	6.5	94.0	0.035	38.9	1
$Zn_{2.92}$/NaSSZ-13 (oxide)	2.92	550	1:1:18	3600	23.0	11.5	93.5	0.048	50.0	6
Zn/Na-SSZ-13 (oxide)	8	550	1:1:18	3600	6.0	5.2	100	0.014	86.7	7
5Mo/5CeTiO$_x$ (oxide)	5	550	1:1:18	6000	15.37	-	72.49	0.042	-	8
Mo_2C	-	600	1:1:2	24000	2.0	1.0	59.5	0.089	50	9
5Fe/10NiMgZr (oxide)	5	600	8.5:8.5:83	3571	23.0	26.5	69.8	0.061	-	10
5Fe/10NiMgZrO$_x$ (oxide)	5	650	8.6:8.6:82.8	7500	23.3	25.5	90.1	0.169	-	11
5KCr/CeZrO$_2$ (oxide)	15	700	1:1:3	6000	32.0	27.3	82.5	0.396	85.3	12
Ni_1Fe_3-CeO_2 (Metallic)	1.93	600	1:1:2	24000	3.5	5.9	77.5	0.203	-	13
NiFe-CeO_2 (Metallic)	19.2	600	1:1:2	24000	4.6	6.2	83.0	0.286	-	14
Fe-β-Mo_2C (Metallic)	1	600	1:1:2	24000	8.7	13.0	62.0	0.405	-	15
Mo_2C/SiO_2	1.42	600	1:1:6	1440	15.5	-	83.5	0.029	-	16
Mo_xC_y/SiO_2	21.4	650	1:1:2	9400	12.5	-	62.0	0.233	-	17

Fe-Mo_xC_y/SiO_2	20.5	600	1:1:2	15000	2.3	-	78.0	0.084	-	[18]
Mo_2C/Al_2O_3	20	600	1:1:2	15000	8.3	5.7	62.0	0.241	68.7	[19]

Supplementary Note:

1. The performance summarization is limited within reaction temperature of ≤700 ºC and CO_2/Ethane ratio of 1;
2. Noble metal-based/contained catalysts are not included;
3. The higher CO_2 conversion than ethane conversion generally results from dry reforming reaction between CO_2 and C_2H_6;
4. The units for the reactants composition column is molar ratio.

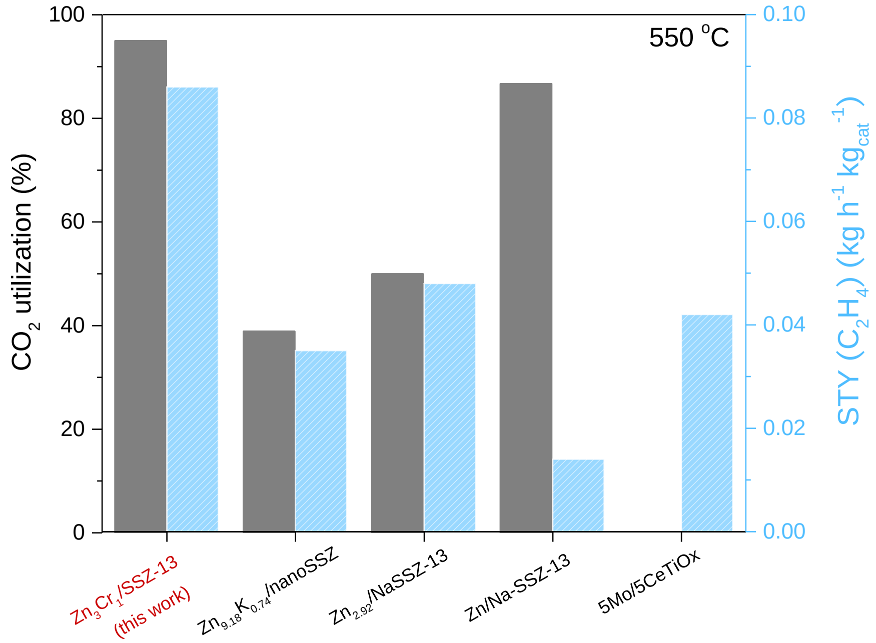

Supplementary Fig. 21. Activity comparisons with reported oxides catalysts at CO_2/C_2H_6 ratio of 1 (550 °C).

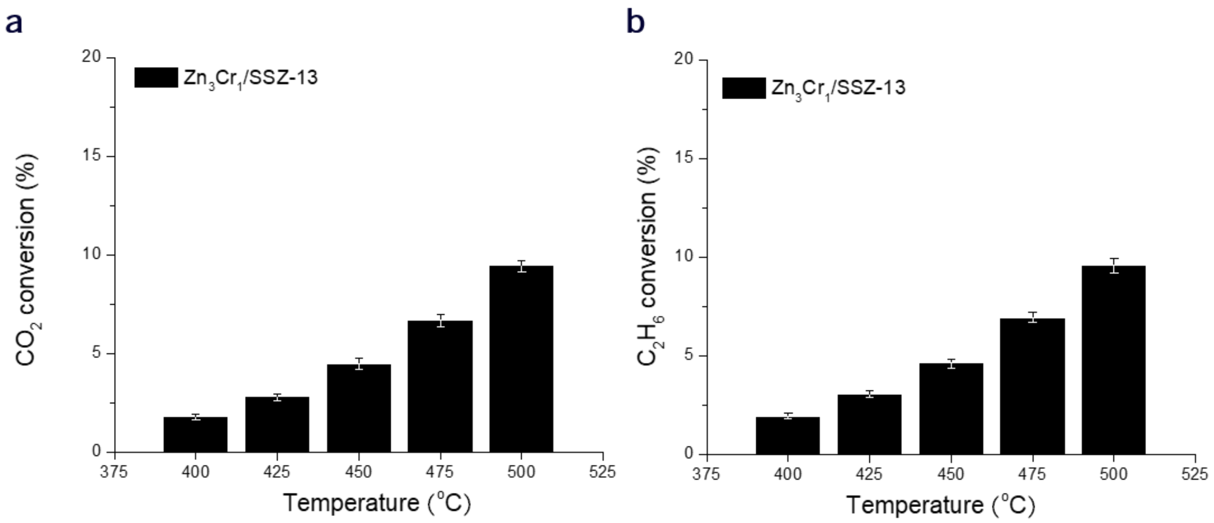

Supplementary Fig. 22. a, CO_2 and **b**, C_2H_6 conversion versus temperatures (within 10% conversion) for Zn_3Cr_1/SSZ-13.

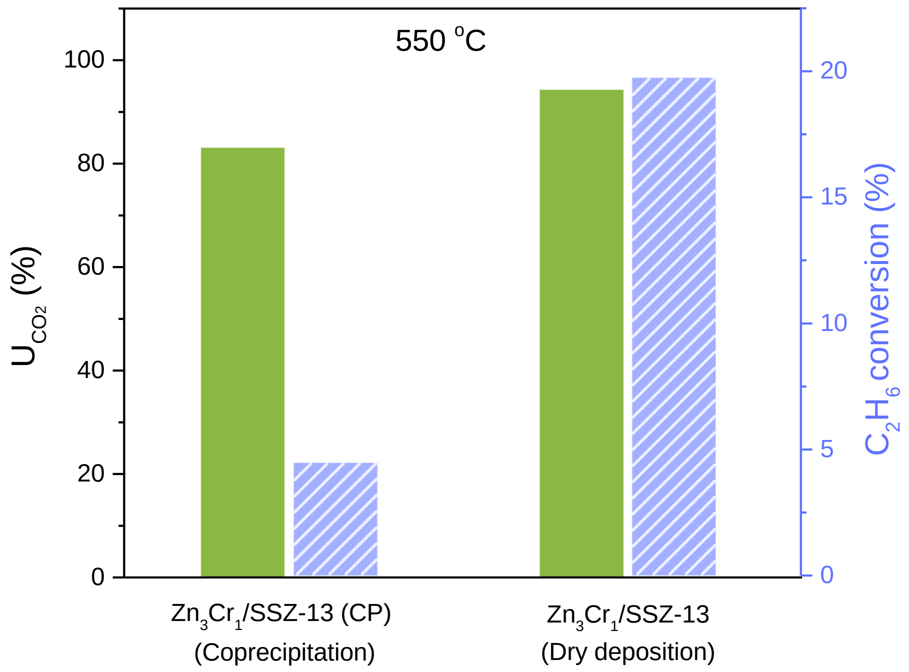

Supplementary Fig. 23. The U_{CO_2} and ethane conversion on Zn_3Cr_1/SSZ-13 catalysts prepared by dry-deposition and co-precipitation (CP) methods.

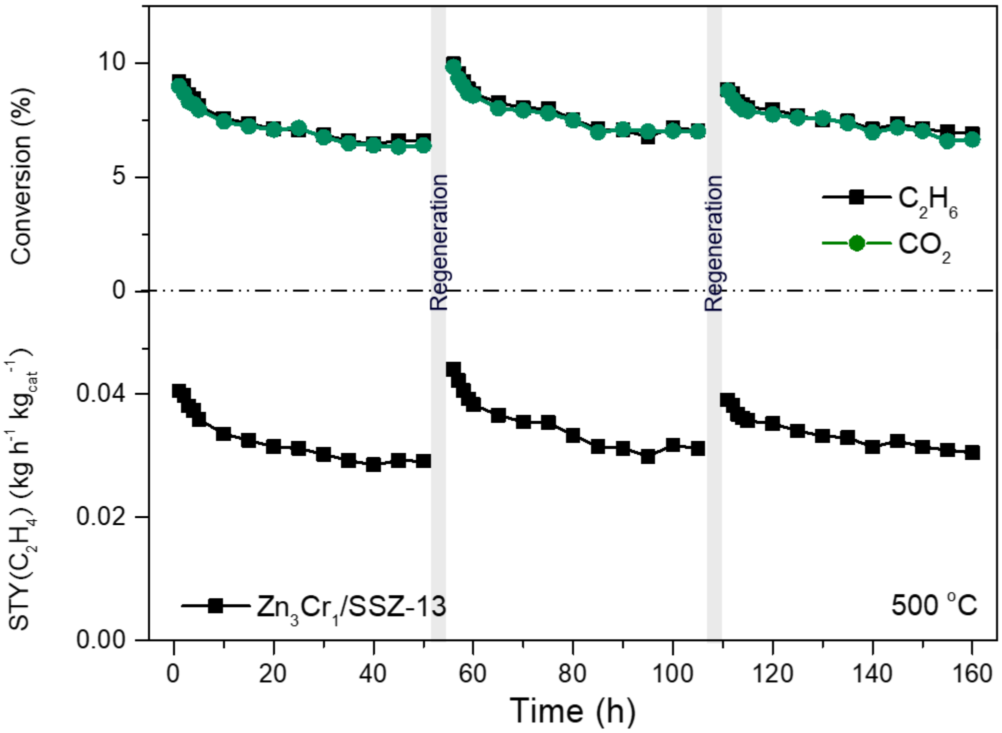

Supplementary Fig. 24. On-stream curves of C_2H_6, CO_2 conversion & the space time yield (STY) of C_2H_4 formation over Zn_3Cr_1/SSZ-13 ABC catalyst.

The practical application potential of Zn_3Cr_1/SSZ-13 ABC for active, selective, durable, and reusable iso-stoichiometric co-conversion of ethane and CO_2 (ICEC) was examined in a series of tests consisting of cycles of ICEC (50h on stream, 500 ^oC) & regeneration (air, 5h, 500 ^oC). After 50 hours of the 1st cycle, the conversion of ethane and CO_2 decreased by ~25%. And STY of ethylene decreased from ~0.042 to ~0.032 kg h^{-1} kg$_{cat}^{-1}$, while maintaining ~100% selectivity for ethylene production. Besides, the decayed conversion of reactants and STY of ethylene can be completely restored by regeneration treatment in air at 500 ^oC. The observed deactivation in the initial induction period should result from the part of loss of the atomic synergies of the $Zn^{\delta+}$-O-Cr^{6+} site (See Supplementary Figs. 25-28).

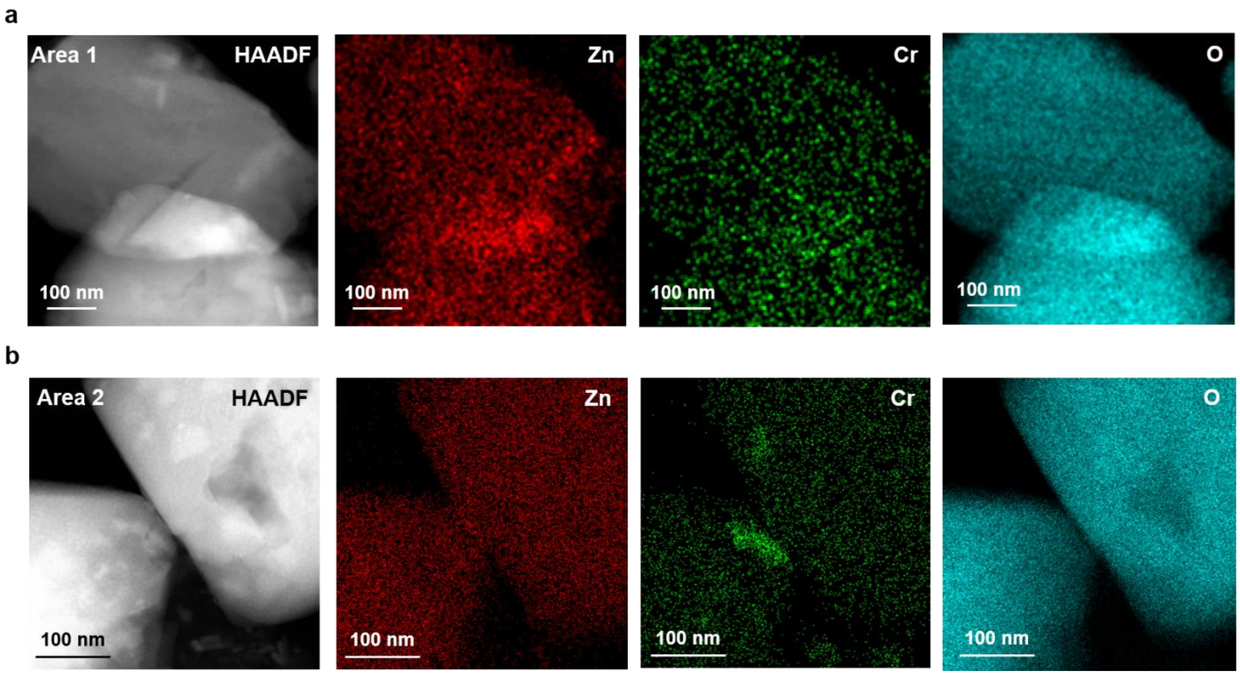

Supplementary Fig. 25. Representative TEM images in different areas of used Zn_3Cr_1/SSZ-13 catalyst after stability test: **a**, Area 1; **b**, Area 2.

As shown in TEM image in the Supplementary Fig. 25, the sintering of ZnO and CrOx was observed after stability tests.

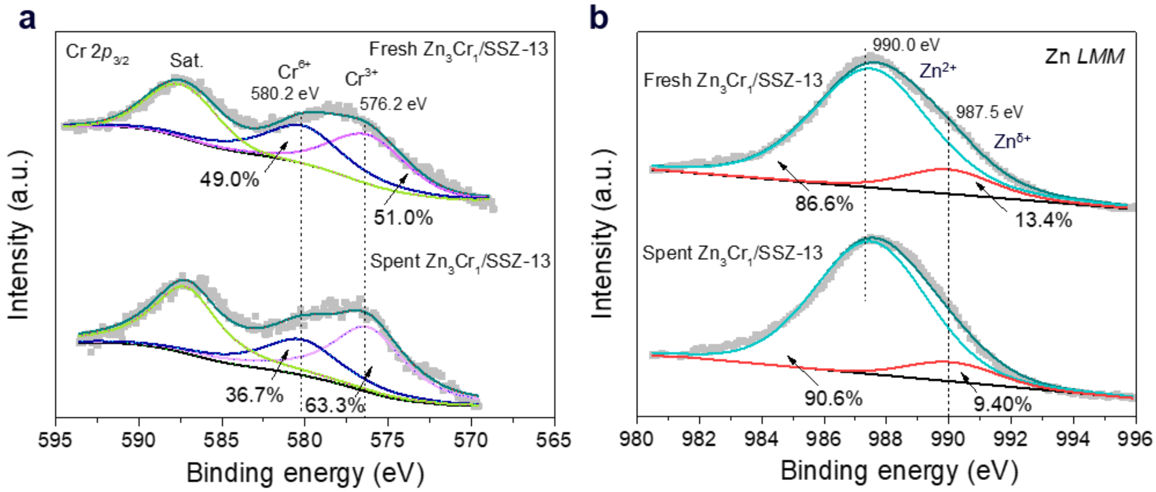

Supplementary Fig. 26. **a**, Cr 2*p* XPS and **b**, Zn *LMM* Auger spectra for fresh and used Zn_3Cr_1/SSZ-13 catalyst after stability test.

XPS results in Supplementary Fig. 26 show that the relative ratios of Zn^{2+} is increased and the relative ratio of Cr^{6+} is decreased for spent catalyst compared to fresh catalyst (Zn^{2+}: 90.6%% vs.86.6%; Cr^{6+}: 36.7% vs. 49.0%). Therefore, we think the part of loss of the atomic synergies of the $Zn^{\delta+}$-O-Cr^{6+} site results in the slight deactivation of Zn_3Cr_1/SSZ-13 catalyst during ICEC reaction.

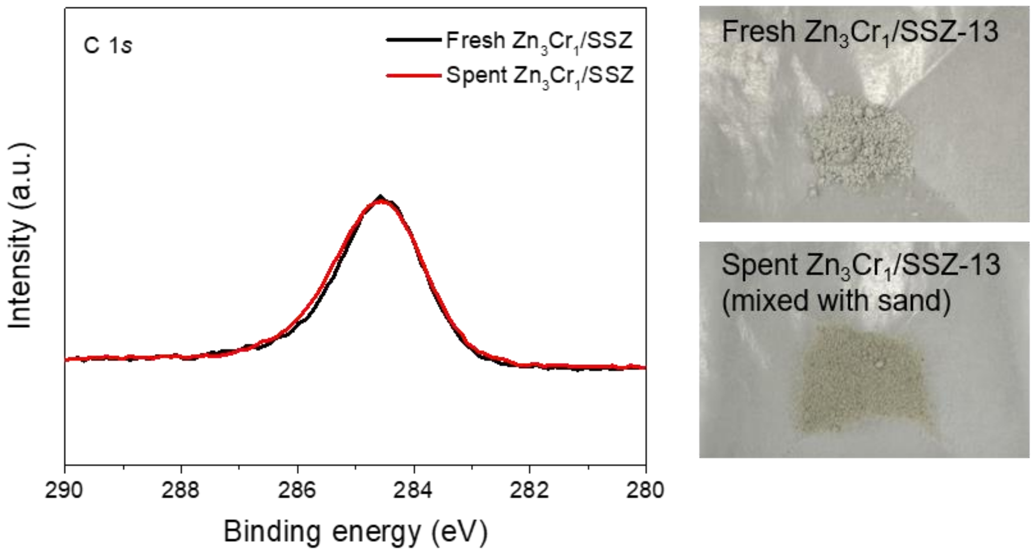

Supplementary Fig. 27. C 1*s* XPS spectra for fresh and spent Zn_3Cr_1/SSZ-13 catalyst.

As shown in Supplementary Fig. 27, the intensity of C 1*s* XPS spectra for spent Zn_3Cr_1/SSZ-13 is similar to that of fresh catalyst. This indicates that carbon deposition on Zn_3Cr_1/SSZ-13 is negligible during ICEC reaction at working conditions. A change of color from an initial light gray to light green yellow (chartreuse) for catalyst powder after stability tests also excludes the carbon deposition during ICEC reaction.

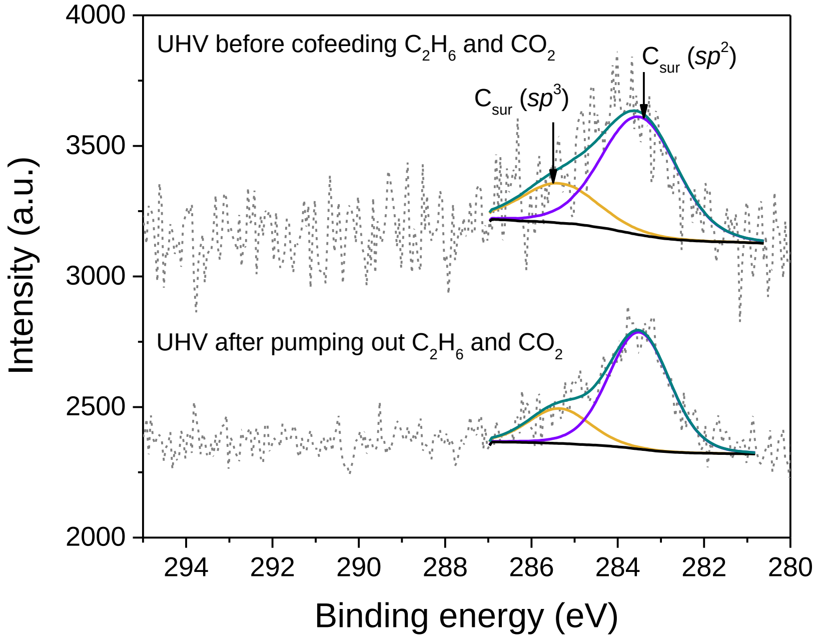

Supplementary Fig. 28. In situ ambient pressure X-ray photoelectron spectroscopy (APXPS): C 1*s* spectra in UHV before cofeeding C_2H_6 and CO_2 and in UHV after pumping out C_2H_6 and CO_2.

As shown in Supplementary Fig. 28, in ultra-high vacuum (UHV) condition before cofeeding C_2H_6 and CO_2, very small amount of surface carbon species (sp^2 and sp^3 carbon) were observed on fresh Zn_3Cr_1/SSZ-13, which is comparable to that in UHV after pumping out C_2H_6 and CO_2. This suggests that the accumulation of carbon species on catalyst surface is negligible during ICEC reaction.

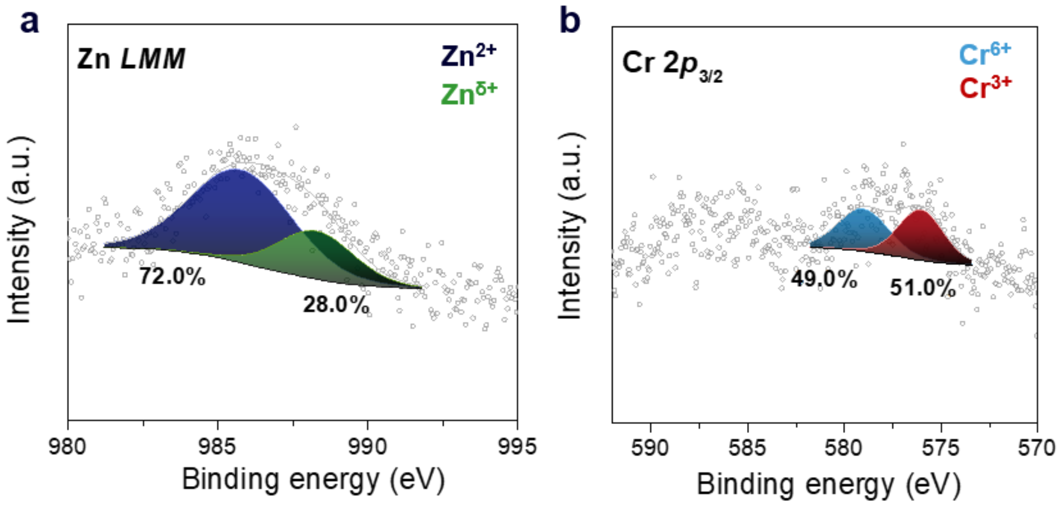

Supplementary Fig. 29. *In situ* ambient pressure X-ray photoelectron spectroscopy (APXPS): **a**, Auger spectra of Zn *LMM*, and **b**, Cr 2$p_{3/2}$ spectra for Zn_3Cr_1/SSZ-13 upon re-exposure to UHV after pumping out C_2H_6 and CO_2. T = 550 ^{o}C.

As shown in supplementary Fig. 29, once that the catalyst was re-exposed to UHV after pumping out C_2H_6 and CO_2, the $Zn^{\delta+}$ ratio was recovered to 28.0% and Cr^{6+} ratio was recovered to 49.0%, which is similar to the initial state of the catalyst (Fig. 3). This is a further evidence that binuclear $Zn^{\delta+}$-O-Cr^{6+} sites are dynamically stable in catalysis.

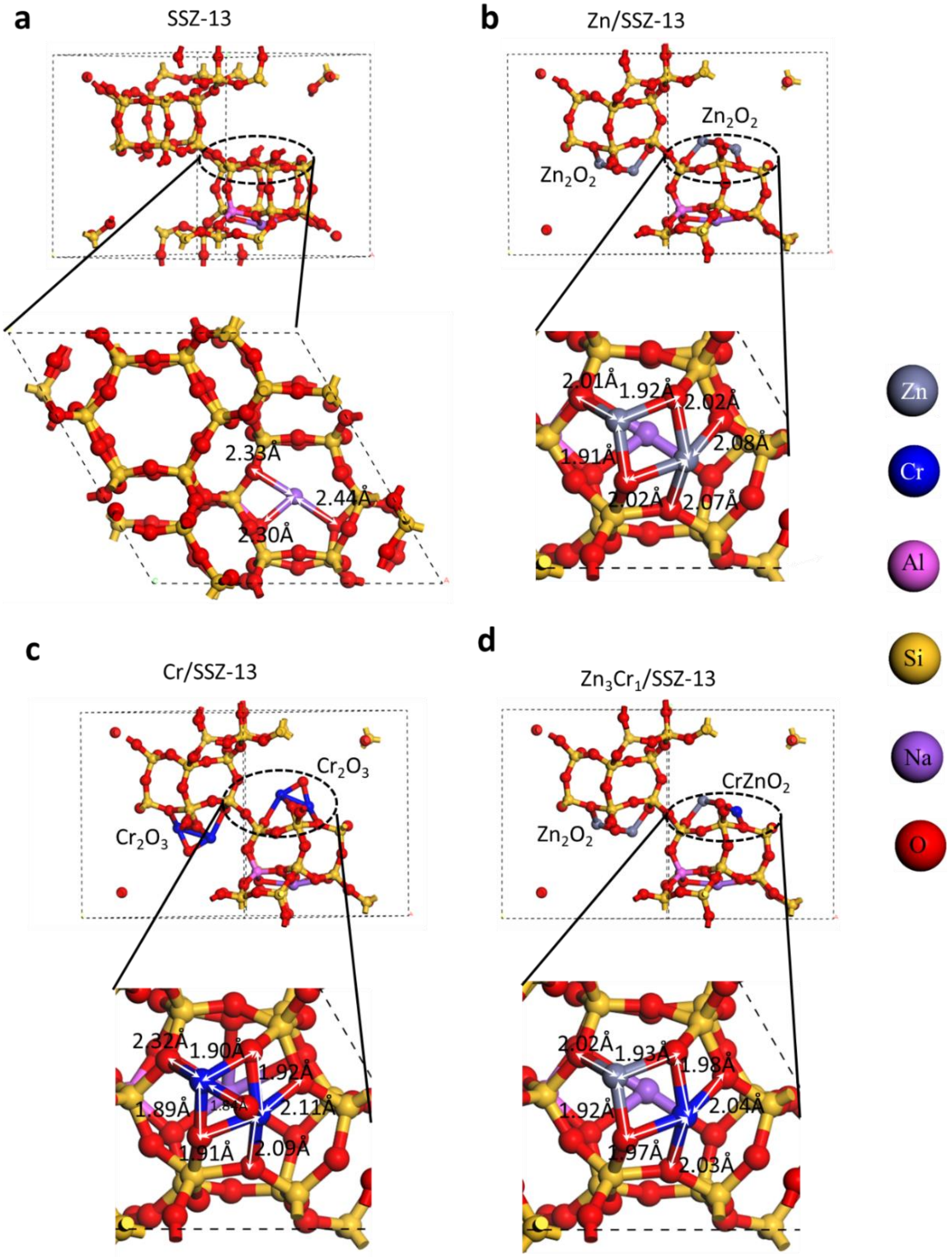

Supplementary Fig. 30. Geometric structure of **a**, SSZ-13, **b**, Zn/SSZ-13, **c**, Cr/SSZ-13, and **d** Zn_3Cr_1/SSZ-13 DFT models.

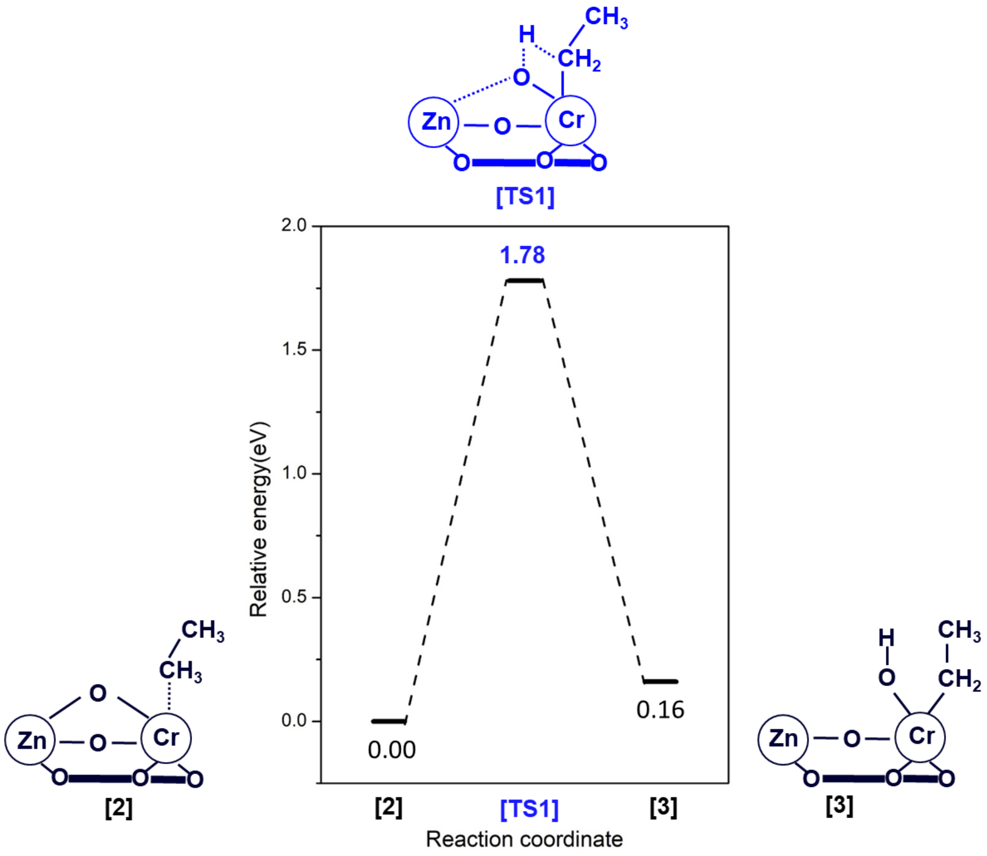

Supplementary Fig. 31. The activation energy barrier of the 1st C-H bond scission in ethane at Cr site on Zn_3Cr_1/SSZ-13.

The energy barrier for the cleavage of the 1st C-H bond in ethane at Cr is 1.78 eV, which is much higher than that at Zn site (0.93 eV).

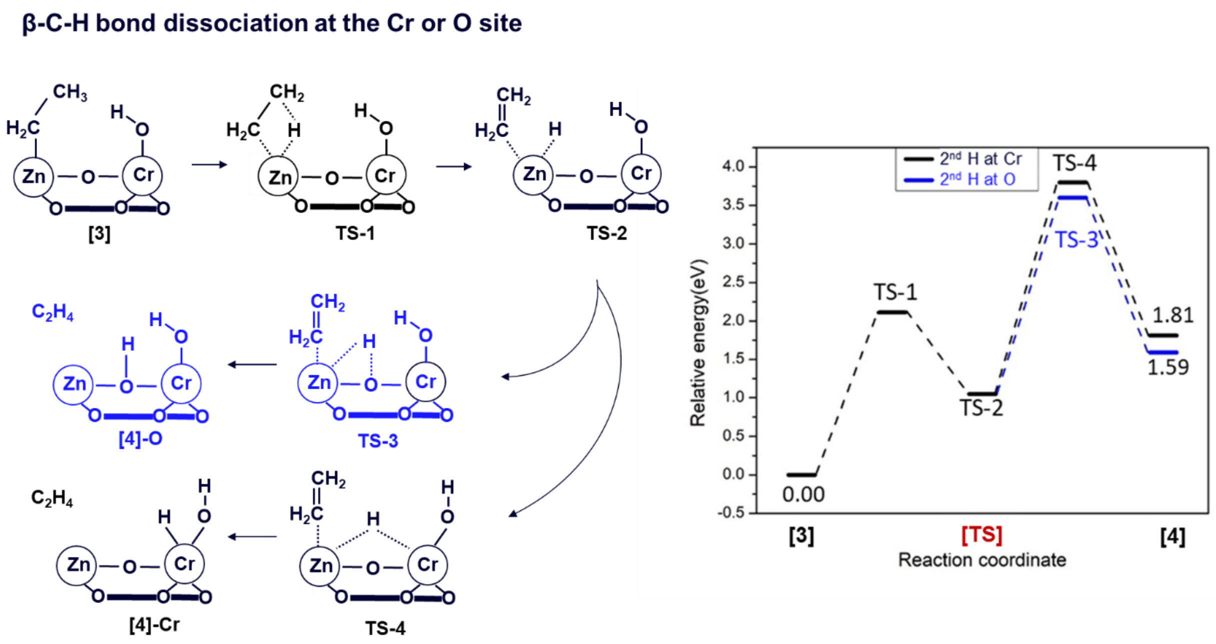

Supplementary Fig. 32. The scenario of β-C-H bond dissociation in ethane by Cr site or O site on Zn_3Cr_1/SSZ-13.

The activation energy barriers (>3 eV) for β-C-H bond dissociation by Cr site or O site are much higher than that at Zn site (2.11 eV), indicating its unfavorable energetics.

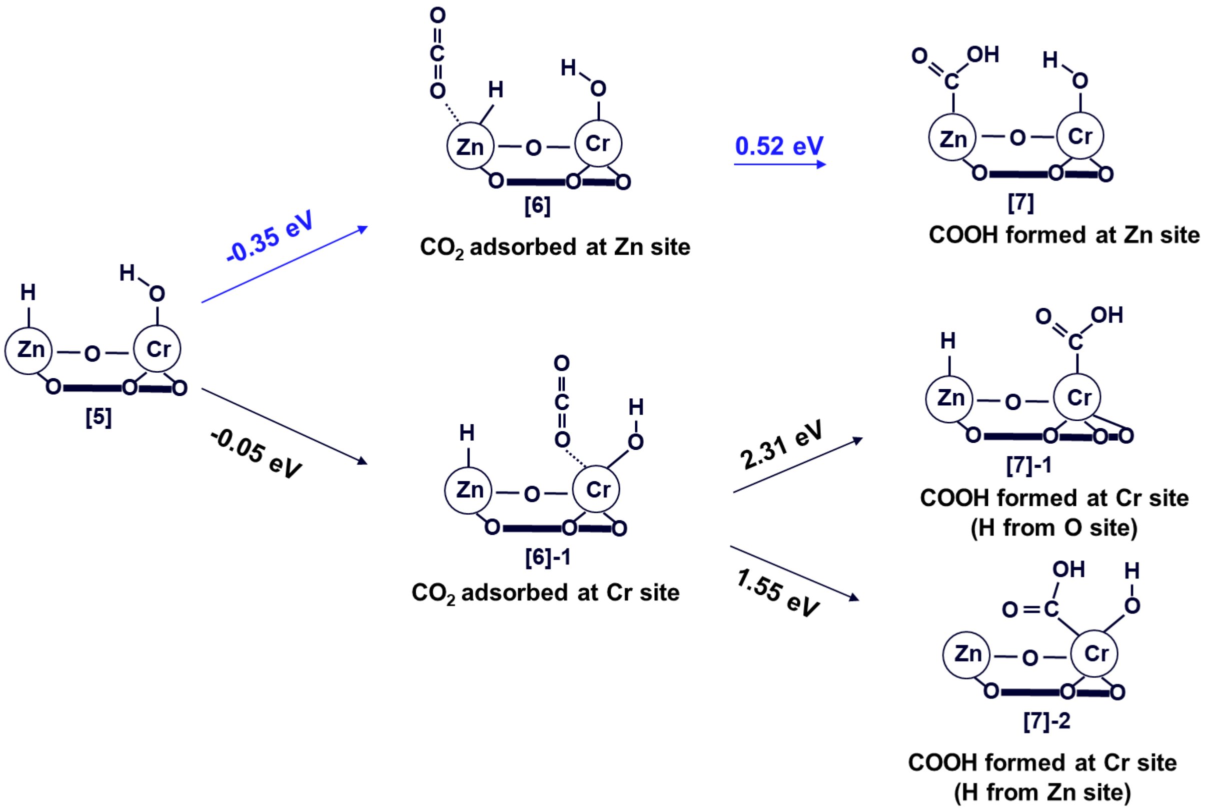

Supplementary Fig. 33. The energy of CO_2 adsorption and activation at Zn-H sites and Cr site of Zn_3Cr_1/SSZ-13.

CO_2 adsorption and activation are more thermodynamically favorable at Zn-H sites than those at Cr site.

Supplementary Table 8. Adsorption energy of CO_2 and the 2nd C_2H_6 at Zn-H hydride of Zn_3Cr_1/SSZ-13 model.

Molecules	CO_2	The 2nd C_2H_6
Adsorption energy (eV)	-0.35	-0.17

With a lower adsorption energy, CO_2 is thermodynamically favorably adsorbed at Zn-H hydride site than the 2nd C_2H_6 molecule, which helps prevent C_2H_6 cracking reaction.

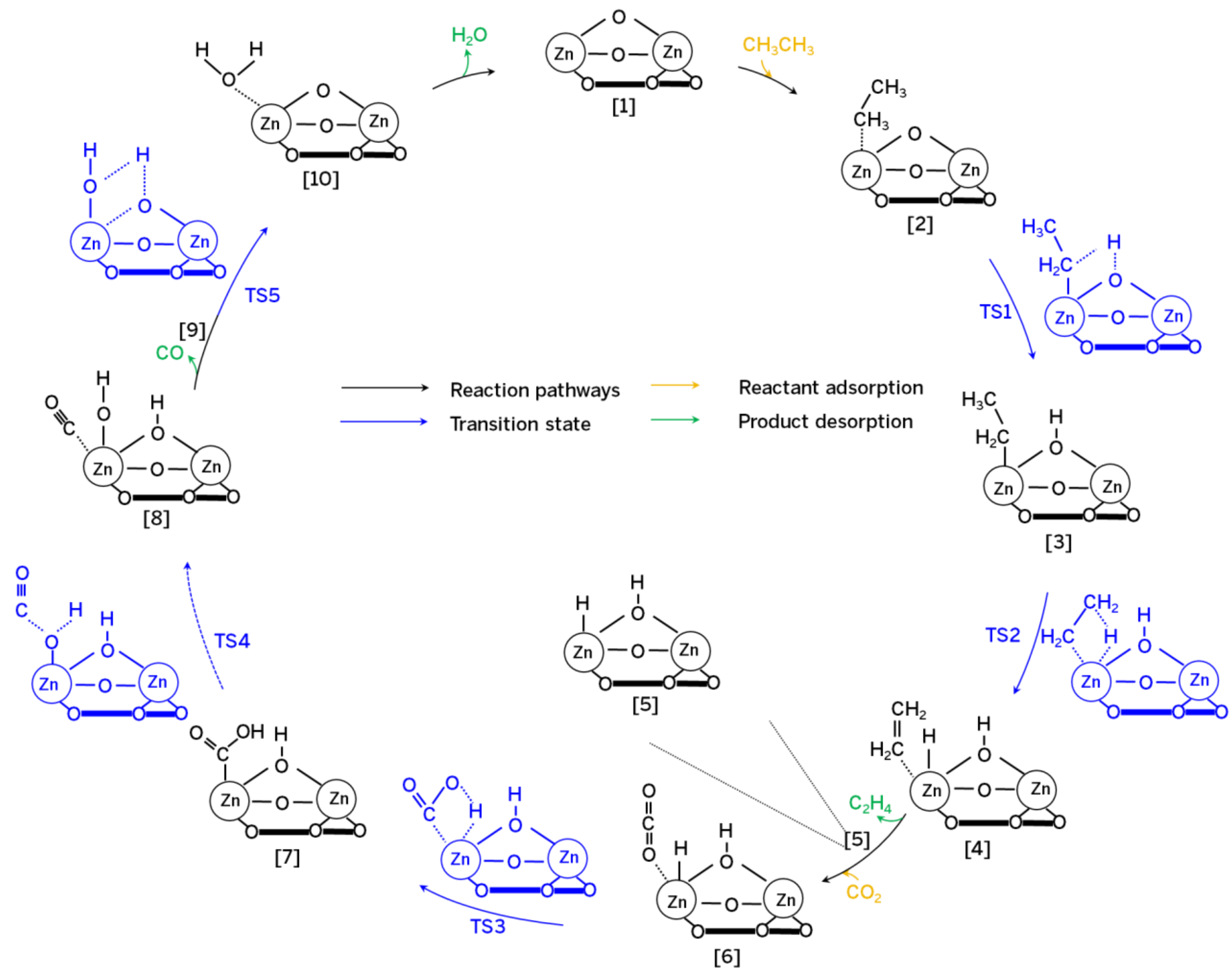

Supplementary Fig. 34. Simulated reaction pathways of iso-stoichiometric co-conversion of ethane and CO_2 on Zn/SSZ-13.

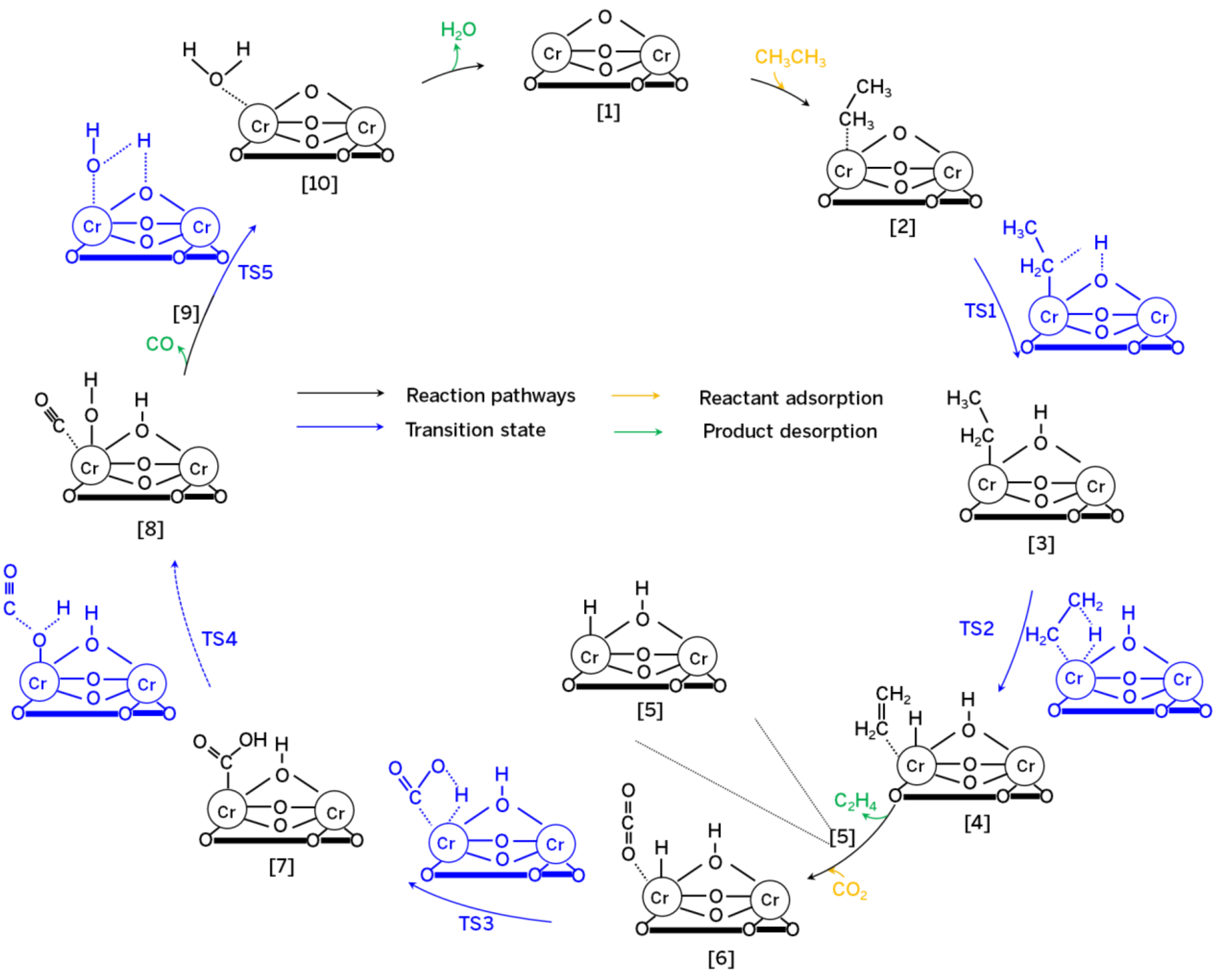

Supplementary Fig. 35. Simulated reaction pathways of iso-stoichiometric co-conversion of ethane and CO_2 on Cr/SSZ-13.

Supplementary Table 9. Energy barriers of different reaction steps in various systems.

	**[TS1]** C_2H_6 → C_2H_5 + H	**[TS2]** C_2H_5 → C_2H_4 + H	**[TS3]** CO_2 + H → COOH	**[TS4]** COOH→ CO + OH	**[TS5]** OH + H → H_2O
Zn_3Cr_1/SSZ-13	0.93	2.11	1.12	0.87	0.73
Zn/SSZ-13	0.92	2.37	1.53	0.55	0.94
Cr/SSZ-13	0.99	2.23	1.08	0.92	0.81

The energies of the transition states in the three different systems are listed in Supplementary Table 9. It can be found the C_2H_5 dehydrogenation (β-C-H bond dissociation) step (**[TS2]**) is the rate-determining step of the reaction for Zn/SSZ-13, Cr/SSZ-13, and Zn_3Cr_1/SSZ-13 systems. All transition states are up to above 2 eV. Compared to Zn/SSZ-13, the co-presence of Cr in Zn_3Cr_1/SSZ-13 decreased energy barrier of **[TS2]**. Besides, Zn-Cr synergy in Zn_3Cr_1/SSZ-13 system significantly reduced energy barrier (1.12 eV) of CO_2 activation (**[TS3]**) compared to Zn/SSZ-13 (1.53 eV). In addition, Zn-Cr synergy facilitates recoupling of OH and H to form H_2O **[TS5]**.

Supplementary Table 10. Reactants (C_2H_6 and CO_2) adsorption energy and products (C_2H_4, CO, and H_2O) desorption energy in various systems.

	C_2H_6 adsorption	CO_2 adsorption	C_2H_4 desorption	CO desorption	H_2O desorption
Zn_3Cr_1/SSZ-13	-0.84	-0.35	0.37	0.94	0.56
Zn/SSZ-13	-0.24	-0.08	0.53	1.12	0.75
Cr/SSZ-13	-0.28	-0.28	0.81	0.91	0.64

By comparing the reaction paths in these three catalyst systems in Fig. 4b and Supplementary Table 10, it is obvious that Zn_3Cr_1/SSZ-13 exhibits a better ability to adsorb C_2H_6 & CO_2 and desorb C_2H_4, CO & H_2O than Zn/SSZ-13 and Cr/SSZ-13, which will help accelerate catalytic cycle.

References

1. Liu, J. *et al.* Influence of the zeolite surface properties and potassium modification on the Zn-catalyzed CO_2-assisted oxidative dehydrogenation of ethane. *Appl. Catal. B Environ.* **304**, 120947 (2022).

2. Zhao, D. *et al.* In situ formation of ZnO_x species for efficient propane dehydrogenation. *Nature* **599**, 234–238 (2021).

3. Dadlani, A. *et al.* Revealing the Bonding Environment of Zn in ALD Zn(O,S) Buffer Layers through X-ray Absorption Spectroscopy. *ACS Appl. Mater. Interfaces* **9**, 39105–39109 (2017).

4. Bernasconi, L., Baerends, E. J. & Sprik, M. Long-range solvent effects on the orbital interaction mechanism of water acidity enhancement in metal ion solutions: A comparative study of the electronic structure of aqueous Mg and Zn dications. *J. Phys. Chem. B* **110**, 11444–11453 (2006).

5. Qiao, L. *et al.* The impact of crystal symmetry on the electronic structure and functional properties of complex lanthanum chromium oxides. *J. Mater. Chem. C* **1**, 4527–4535 (2013).

6. Liu, J. *et al.* Highly-Dispersed Zinc Species on Zeolites for the Continuous and Selective Dehydrogenation of Ethane with CO_2 as a Soft Oxidant. *ACS Catal.* **11**, 2819–2830 (2021).

7. Jiang, Y. *et al.* Preparation of Shaped Binder-Free SSZ-13 Zeolite and Its Application in CO_2 Adsorption and Catalysis. *ChemCatChem* **14**, e202200795 (2022).

8. Nguyen, T. D., Zheng, W., Celik, F. E. & Tsilomelekis, G. CO_2-assisted ethane oxidative dehydrogenation over MoO: Xcatalysts supported on reducible CeO_2-TiO_2. *Catal. Sci. Technol.* **11**, 5791–5801 (2021).

9. Porosoff, M. D. *et al.* Identifying Different Types of Catalysts for CO_2 Reduction by Ethane through Dry Reforming and Oxidative Dehydrogenation. *Angew. Chem. Int. Ed* **54**, 15501–15505 (2015).

10. Theofanidis, S. A., Kasun Kalhara Gunasooriya, G. T., Itskou, I., Tasioula, M. & Lemonidou, A. A. On-purpose Ethylene Production via CO_2-assisted Ethane Oxidative Dehydrogenation: Selectivity Control of Iron Oxide Catalysts. *ChemCatChem* **14**, e202200032 (2022).

11. Tasioula, M. *et al.* Tandem CO_2 Valorization and Ethane Dehydrogenation: Elucidating the Nature of Highly Selective Iron Oxide Active Sites. *ACS Catal.* **13**, 2176–2189 (2023).

12. Li, Y. *et al.* The role of K in tuning oxidative dehydrogenation of ethane with CO_2 to be selective toward ethylene. *Adv. Compos. Hybrid Mater.* **4**, 793–805 (2021).

13. Yan, B. *et al.* Active sites for tandem reactions of CO_2 reduction and ethane dehydrogenation. *Proc. Natl. Acad. Sci.* **115**, 8278–8283 (2018).

14. Guo, M., Feng, K., Wang, Y. & Yan, B. Unveiling the Role of Active Oxygen Species in Oxidative Dehydrogenation of Ethane with CO_2 over NiFe/CeO_2. *ChemCatChem* **13**, 3119–3131 (2021).

15. Yao, S. *et al.* Combining CO_2 Reduction with Ethane Oxidative Dehydrogenation by Oxygen-Modification of Molybdenum Carbide. *ACS Catal.* **8**, 5374–5381 (2018).

16. Solymosi, F. & Németh, R. The oxidative dehydrogenation of ethane with CO_2 over Mo_2C/SiO_2 catalyst. *Catal. Lett.* **62**, 197–200 (1999).

17. Marquart, W., Claeys, M. & Fischer, N. CO_2 reduction and C_2H_6 dehydrogenation over SiO_2 supported molybdenum carbide nanoparticles. *Appl. Catal. A Gen.* **663**, 119291 (2023).

18. Marquart, W., Raseale, S., Claeys, M. & Fischer, N. Promoted Mo_xC_y-based Catalysts for the CO_2 Oxidative Dehydrogenation of Ethane. *ChemCatChem* **14**, (2022).

19. Marquart, W., Claeys, M. & Fischer, N. Conversion of CO_2 and small alkanes to platform chemicals over Mo2C-based catalysts. *Faraday Discuss.* **230**, 68–86 (2021).
